# Model-assisted DoE software: optimization of growth and biocatalysis in *Saccharomyces cerevisiae* bioprocesses

**DOI:** 10.1007/s00449-020-02478-3

**Published:** 2021-01-20

**Authors:** André Moser, Kim B. Kuchemüller, Sahar Deppe, Tanja Hernández Rodríguez, Björn Frahm, Ralf Pörtner, Volker C. Hass, Johannes Möller

**Affiliations:** 1 Faculty of Medical and Life Sciences, Furtwangen University of Applied Sciences, Villingen-Schwenningen, Germany; 2 Institute of Bioprocess and Biosystems Engineering, Hamburg University of Technology, Hamburg, Germany; 3 Biotechnology and Bioprocess Engineering, Ostwestfalen-Lippe University of Applied Sciences and Arts, Lemgo, Germany

**Keywords:** Biocatalysis, Monte Carlo methods, Fed-batch strategy, Model-assisted design of experiments, Quality by design

## Abstract

Bioprocess development and optimization are still cost- and time-intensive due to the enormous number of experiments involved. In this study, the recently introduced model-assisted Design of Experiments (mDoE) concept (Möller et al. in Bioproc Biosyst Eng 42(5):867, 10.1007/s00449-019-02089-7, 2019) was extended and implemented into a software (“mDoE-toolbox”) to significantly reduce the number of required cultivations. The application of the toolbox is exemplary shown in two case studies with *Saccharomyces cerevisiae*. In the first case study, a fed-batch process was optimized with respect to the pH value and linearly rising feeding rates of glucose and nitrogen source. Using the mDoE-toolbox, the biomass concentration was increased by 30% compared to previously performed experiments. The second case study was the whole-cell biocatalysis of ethyl acetoacetate (EAA) to (*S*)-ethyl-3-hydroxybutyrate (E3HB), for which the feeding rates of glucose, nitrogen source, and EAA were optimized. An increase of 80% compared to a previously performed experiment with similar initial conditions was achieved for the E3HB concentration.

**Supplementary Information:**

The online version contains supplementary material available at 10.1007/s00449-020-02478-3.

## Introduction

Biotechnology is expected to make a significant contribution to the establishment of a bio-based economy, since it offers new product manufacturing approaches and resource-efficient technologies [2, 3]. However, the development of a bio-economy requires new sustainable and environmentally friendly industrial production processes [4, 5]. Experiments for their development and optimization are usually designed using one-factor-at-a-time approaches and statistical Design of Experiments (DoE) methods. DoE methods inevitably require a large number of experiments to be performed and analytically evaluated [6, 7]. Although the use of high-throughput systems is well established, e.g., for the screening of new enzymes or drugs [8, 9], they can be used with simplifications only for the actual process development (e.g., dimensioning of bioreactors, design of process control strategies, and scale-up) [10–12]. Although DoE can be used to identify correlations between process parameters and their influence on the final productivity, the complex bioprocess is reduced to a few key numbers (e.g., final product concentration), and the dynamics of growth and metabolism are not sufficiently taken into account [1, 13, 14]. In addition, the heuristic conception of a DoE by choosing the limits of the parameter space poses a particular challenge [15–17]. Thus, there is a high risk that the experiments carried out were wrongly chosen and have only insufficient validity, which results in further costs and time delays [1, 18].

To overcome the previously mentioned limitations of DoE, a new model-assisted Design of Experiments (mDoE) concept was recently introduced for knowledge-driven bioprocess development and optimization [1, 14, 19]. In the mDoE approach, the recommended experiments in statistical DoE designs are simulated using mathematical process models instead of being performed experimentally. The DoE designs (i.e., experimental space) are then evaluated based on the simulations, which enables the definition of a well-defined experimental space with a significantly reduced number of experiments to be performed experimentally. Besides the significant reduction of the number of experiments, the use of mathematical process models is nowadays seen as a sustainable part of a knowledge-driven bioprocess development strategy, because they contribute to the scientific understanding of the process [12, 20–22]. So far, the successful application of the mDoE approach in the field of medium and feeding strategy optimization for an antibody-producing Chinese Hamster Ovary cell line was shown [1, 14, 19]. Among the here presented application in the field of bio-economy, mDoE is currently used in optimization studies with algae, stem cells, and different mammalian producer cell lines.

In this study, the mDoE concept is incorporated into a software toolbox (“mDoE-toolbox”) for efficient design and optimization of biotechnological processes with a reduced number of experiments. In general, the toolbox can be used for different applications such as cell culture, algae, and yeast. Here, the application of the mDoE-toolbox is shown in two optimization case studies with *Saccharomyces cerevisiae*. In the first case study (S1), the cultivation conditions of a fed-batch process were optimized to increase the biomass concentration. The chosen factors were the pH value of the medium and the feeding rates of linearly rising feeding rates for glucose ($F_{\mathrm{Glc}}$) and nitrogen source ($F_{\mathrm{N}}$). In the second case study (S2), the concentration of (*S*)-ethyl-3-hydroxybutyrate (E3HB) was maximized in the biocatalytic conversion of ethyl acetoacetate (EAA) to E3HB based on constant feeding rates for EAA ($F_{\mathrm{EAA}}$), glucose ($F_{\mathrm{Glc}}$), and nitrogen source ($F_{\mathrm{N}}$).

### mDoE-toolbox

The mDoE concept (see [1, 14]) was incorporated into a software toolbox, implemented in MATLAB (V2018a) and R (V3.5.1). The main parts of the mDoE-toolbox are the combination of a mathematical process model, including model-parametric uncertainties with the computational planning and evaluation of DoE designs.

**Fig. 1:**
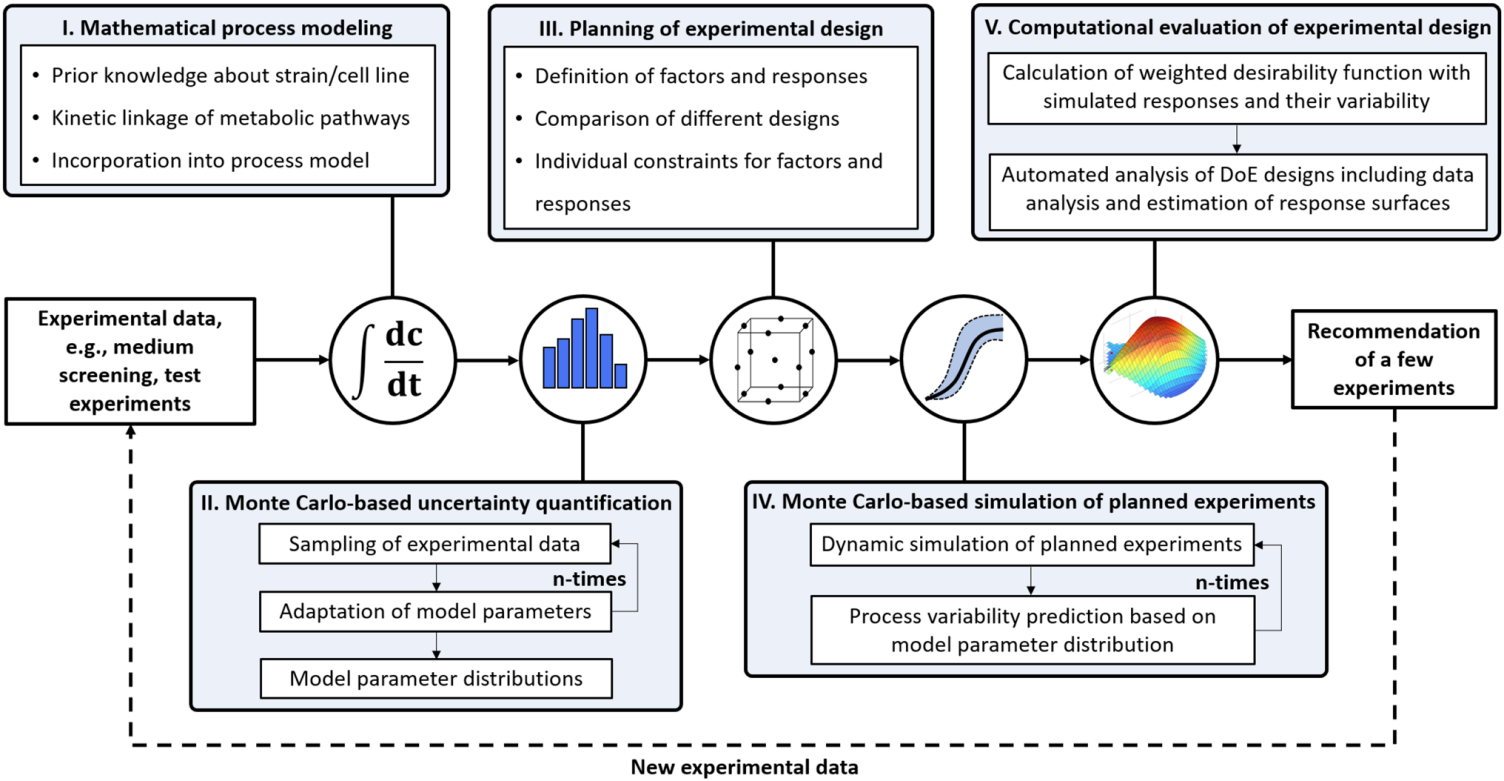
Structural workflow of mDoE-toolbox consisting of the combination of mathematical process models and classical DoE under the consideration of model-parametric uncertainty based on experimental variability [1, 19]

In the beginning, the objective of the study (i.e., maximization of product concentration and minimization of inhibitory component) is defined. Then, the biotechnological system is modeled first, as can be seen in the structural workflow in Fig. 1 box I. Thus, prior knowledge (e.g., pre-experiments and literature) about the strain is used to define mathematical expressions for cell growth, metabolism, and productivity [1]. It should be noticed that process modeling itself is a rather undefined work, and a variety of models and modeling approaches of different considered complexity exist in the literature [24–25]. The mDoE-toolbox is designed to be applied in the initial phase of process development for which very little data are available. Therefore, structurally simple [14, 26] or generalized models [27] are applied, for which model parameters can be adapted based on few data points typically generated in medium tests or first cultivations. After defining a mathematical model, the model-parametric uncertainties are derived with Monte Carlo sampling based on the experimental uncertainty (i.e., measurement error, Fig. 1 box II). Therefore, the expected process variability based on the measurement errors is simulated and later used in the DoE evaluation [19, 28]. Next, the experimental factors and responses are defined (Fig. 1 box III) with individual boundary values, e.g., a tolerated concentration of an inhibitory component or a minimal required product concentration. A DoE design, such as an optimal design [29, 30] or Box–Behnken design [31, 32], is subsequently planned. Additionally, mDoE enables the *in silico* comparison of different DoE designs, which is not targeted in this study.

For each recommended factor combination *i*, the time courses of the modeled state variables (e.g., cell weight, substrate, and product concentration) are simulated multiple times (Monte Carlo simulations, Fig. 1 box IV) taking into account the previously determined parameter probability functions (box II). From these simulations, the average expected response ${\overline{r}}_{i}$ (e.g., average maximal cell dry weight) and the variability $\nu _{i}$ are calculated. Due to Monte Carlo simulations, $\nu _{i}$ of the response is expressed as the difference between the 10% and 90% quantiles of simulations [19].

In the next step, ${\overline{r}}_{i}$ and $\nu _{i}$ are used for the computational evaluation (box V) of the former planned experimental design (box III). Therefore, both ${\overline{r}}_{i}$ and $\nu _{i}$ are summarized into a combined objective/desirability function (desirability at experimental factor combinations *i*-$D_{i}$) for each planned experiment in the DoE designs [33, 34]. This enables an evaluation of each planned experiment with respect to its simulated average and its expected variability with the aim of simultaneously maximizing ${\overline{r}}_{i}$ and reducing $\nu _{i}$. The evaluation of DoEs using $D_{i}$ reflects a risk-based approach. An experiment with a high $D_{i}$ is favorable and a low $D_{i}$ indicates a high variability and/or a low average response, which is not desired. After calculating $D_{i}$ for all planned and simulated experiments, the experimental design planned in box III is analyzed, and response surface (RS) plots are generated automatically for visualization. Only a few (e.g., 2–4) experiments with the highest $D_{i}$ are recommended to be performed, experiments with low $D_{i}$ are neglected, which enables a significantly reduced number of experiments compared to the initially planned DoE design (box II).

Using the mDoE-toolbox, the available knowledge can be captured in the mathematical model, which can serve as a basis for advanced process understanding and digital twins [36–37]. In this way, the new data obtained from the recommended experiments can be used to re-adapt the model parameters and their probability distribution or to modify the model structure if so far unknown effects were identified [19, 24, 28].

## Materials and methods

### S1: optimization of fed-batch strategy for maximization of dry cell weight

Genetically unmodified *Saccharomyces cerevisiae* (Agrano, Germany, commercial strain used for industrial food production) was cultivated using complex media consisting of water, glucose (Glc), yeast extract (YE), and soy peptone (Pep, all Roth, Germany). No preculture was carried out, and dried yeast was directly inoculated. An overview of the performed experiments in S1 with the medium and feed compositions used is shown in Table 1.

**Table 1:** Initial and feed volume, as well as initial and feed concentration of $c_{\mathrm{DCW}}$, $c_{\mathrm{Glc}}$, $c_{\mathrm{YE}}$ and $c_{\mathrm{Pep}}$ of every experiment in S1

	Medium					$F_ {\mathrm{Glc}}$	$F_ {\mathrm{N}}$	
	Volume (l)	Biomass ($\mathrm{g~l}^{-1}$)	$c_{\mathrm{Glc}}$ ($\mathrm{g~l}^{-1}$)	$c_{\mathrm{YE}}$ ($\mathrm{g~l}^{-1}$)	$c_{\mathrm{Pep}}$ ($\mathrm{g~l}^{-1}$)	$c_{\mathrm{Glc}}$ ($\mathrm{g~l}^{-1}$)	$c_{\mathrm{YE}}$ ($\mathrm{g~l}^{-1}$)	$c_{\mathrm{Pep}}$ ($\mathrm{g~l}^{-1}$)
Modeling								
Cultivation I	1.00	3.0	6.0	1.4	2.3	290	50.0	76.0
Cultivation II	1.00	3.0	6.0	1.4	2.3	290	50.0	76.0
Cultivation III	0.75	20.0	7.9	1.4	2.3	290	85.0	135
pH-Exp	0.30	10.0	30.0	2.9	4.5	180	17.0	27.0
mDoE-toolbox								
S1	0.70	2.0	15.0	1.4	2.3	400	130	270

#### Experiments for modeling

For the parameterization of the pH-related model part (see Section “Mathematical process model”), four cultivations with different pH were performed in 1 l baffled shake flasks (500 ml working volume, Schott, Germany), which were shaken at 170 rpm (1.9 cm shaking diameter, MaxQ4000, Thermo Fisher Scientific, USA) with initial $c_{\mathrm{DCW}}=10~\mathrm{g~l}^{-1}$. The temperature was controlled at $30^\circ \,\mathrm{C}$. The pH was adjusted initially and maintained manually (pH = 3, 4, 5, 6, respectively) to the desired value. In all experiments, the pH was adjusted using 20 wt% potassium hydroxide solution or 20 wt% phosphoric acid (both VWR, Germany). One feed pulse (glucose and nitrogen source) of 50 ml was added to each flask after 24 h (feed concentration in Table 1).

After adjusting the pH part of the model, further model parameterization was done based on historical data of three bioreactor (2 l working volume, Biostat B, Sartorius, Germany) fed-batch cultivations with different initial concentrations and feed compositions (see Table 1). Gassing was manually set in relation to the state of the process between 1 and 2 vvm (max. 2 $\hbox {l min}^{-1}$), and stirring was held between 500 and 800 rpm to maintain a DO above 10%. The pH was automatically controlled at 5. Temperature was set to $30\,^\circ \mathrm{C}$.

#### Recommended experiments from mDoE-toolbox

For the experiments recommended from the mDoE-toolbox, the initial dry cell weight (DCW) was adjusted to $c_{\mathrm{DCW}}=2\mathrm{~g~l}^{-1}$ and the initial conditions of the complex culture medium were prepared, as shown in Table 1. The recommended feeding strategy for the mDoE experiments was a linearly rising feeding rate starting at $t_{\mathrm{start}}=\mathrm{1~h}$. The final feed volume flow at $t_{\mathrm{end}}=48~\mathrm{h}$ was determined by the mDoE-toolbox for $F_{\mathrm{Glc}}$ and $F_{\mathrm{N}}$ separately. The feed rates were determined using the following function:

1 $$\begin{aligned} F_{\mathrm{k}}(t) = \frac{F_{\mathrm{k, end}}}{t_{\mathrm{end}}} \times (t - t_{\mathrm{start}}). \end{aligned}$$

For $t < t_{\mathrm{start}}$, $F_{\mathrm{k}}(t)$ equals zero.

Linear feed strategies have been chosen instead of exponential strategies, as these are easier to handle and less risky. Slightly too large feed rates resulting from exponential strategies quickly result in over-feeding. The pH was held constant using 20 wt% potassium hydroxide solution or 20 wt% phosphoric acid (both VWR). The temperature was controlled at $30\,^\circ \mathrm{C}$.

### S2: optimization of fed-batch strategy for biocatalysis

Genetically unmodified *Saccharomyces cerevisiae* (Agrano, Germany) served as the whole-cell biocatalyst. In the biocatalysis (S2), the media consist of Glc, YE, Pep, and EAA (all Roth, Germany). The temperature was set at $30^\circ \mathrm{C}$ and pH 5. The pH was controlled with the addition of 20 wt% potassium hydroxide solution or 20 wt% phosphoric acid. The airflow rate was adjusted at 1–2 vvm and stirring rate at 800 rpm to maintain aerobic conditions (DO > 10%). Antifoam was fed when required. The experiments for the biocatalysis part (S2) are shown in Table 2.

**Table 2:** Initial and feed volume, as well as initial and feed concentration of $c_{\mathrm{DCW}}$, $c_{\mathrm{Glc}}$, $c_{\mathrm{YE}}$, and $c_{\mathrm{Pep}}$ of the biocatalysis experiment of reference process S2; EAA is fed as a pure component

	Medium					$F_ {\mathrm{Glc}}$	$F_ {\mathrm{N}}$	
	Volume (l)	Biomass ($\mathrm{g~l}^{-1}$)	$c_{\mathrm{Glc}}$ ($\mathrm{g~l}^{-1}$)	$c_{\mathrm{YE}}$ ($\mathrm{g~l}^{-1}$)	$c_{\mathrm{Pep}}$ ($\mathrm{g~l}^{-1}$)	$c_{\mathrm{Glc}}$ ($\mathrm{g~l}^{-1}$)	$c_{\mathrm{YE}}$ ($\mathrm{g~l}^{-1}$)	$c_{\mathrm{Pep}}$ ($\mathrm{g~l}^{-1}$)
Modeling								
Biocatalysis I	4.0	45.5	0.1	0	0	400	130	270
Biocatalysis II	10.0	78.2	0.1	0	0	400	130	270
mDoE-toolbox								
S2	0.6	40.0	2.0	1.6	2.4	Adjusted individually		

#### Experiments for modeling

Two experiments for model parameterization were performed in 5 l (Biocatalysis I) (BioFlo, Eppendorf, Germany) and 20 l reactor (Biocatalysis II) (BioStat C, Sartorius). Initial and feed conditions are shown in Table 2. The first experiment was an initial test experiment and the second experiment was with a high initial $c_{\mathrm{DCW}}$.

#### Recommended experiments from mDoE-toolbox

The recommended biocatalysis experiments were performed in 1 l stirred bioreactors (Medorex, Germany). Initial dry biomass density (cell dry weight $c_{\mathrm{DCW}}$) of $40~\mathrm{g~l}^{-1}$ was chosen. Constant $F_{\mathrm{Glc}}$, $F_{\mathrm{N}}$, and $F_{\mathrm{EAA}}$ were defined as factors. Feeding started immediately after inoculation. Since the independently predicted feed flow rates for glucose and nitrogen were too low for the available pumps, they were fed together. No online off-gas measurement was performed.

### Analytics

Concentrations of ethanol ($c_{\mathrm{EtOH}}$), $c_{\mathrm{Glc}}$, $c_{\mathrm{EAA}}$, and $c_{\mathrm{E3HB}}$ were quantified with high pressure liquid chromatography using a Rezex ROA column (300 $\times$ 7.8 mm, Phenomenex, USA) and 0.005 $\mathrm{N}$ sulfuric acid as the aqueous mobile phase according to the manufacturer’s protocol. $c_{\mathrm{DCW}}$ was determined by filtrating the medium through cellulose acetate filters (0.45 m, VWR, US) and measuring the weight of the retentate after drying in a moisture analyzer (MA45, Sartorius, Germany). The percentages of oxygen and carbon dioxide in the off-gas were measured via an extractive gas analyzer (Sick, Germany). The respiratory quotient (RQ) is then calculated from the quotient of carbon dioxide produced divided by the oxygen consumed [38, 39]:

2 $$\begin{aligned} {{\mathrm{RQ} = \frac{\mathrm{CO}_{\mathrm{2, produced}}[\mathrm {mol}]}{\mathrm{O}_{\mathrm{2, consumed}}[\mathrm {mol}]}}}. \end{aligned}$$

The pH value of the medium was measured in situ with an amperometric pH Probe (405-DPAS-SC-K8S, Mettler Toledo, US, and EasyFerm Plus PHI S8 225, Hamilton, US). The pH values in the shaking flask experiments were controlled offline with a benchtop pH meter (FiveEasy F20, Mettler Toledo, US). The DO in the biocatalysis experiments was measured with an optical dissolved oxygen (DO) probe (VisiFerm DO ECS 225 H0, Hamilton, US).

### Mathematical process model

A novel structured compartment model, capable of being adapted to different biotechnological expression systems (e.g., bacteria, yeast, fungi, mammalian cell lines), was used to describe yeast growth, metabolism, and biocatalysis [27, 40]. The model was previously introduced by Brüning et al. [27] and is briefly explained in the following. The main part of the model is the segregation of the biomass into six distinct compartments, which are linked and individually described by mathematical equations representing different essential metabolic tasks. The detailed figure of the six model compartments can be found in the Electronic Supplementary Material (ESM) Fig. S1.

The following compartments are considered: an autocatalytically active biomass (Xpri), a product forming (Xp), a biocatalytically inactive (Xi), a structurally active (Xs) and inactive (Xsi), and a dead biomass (Xd) compartment. Biomass synthesis is based on a carbon (SC) and a nitrogen substrate (SN) and biocatalysis is modeled based on an educt (SBC). Furthermore, physicochemical state variables, such as DO, pH, and temperature, have a direct influence on cell metabolism, biomass growth and/or biocatalytic activity [27]. The uptake rates (rS) of the substrates (S) are rate-limiting steps, which are modeled by Monod kinetics typically used in bioprocess modeling [14, 26, 28, 40]:

3 $$\begin{aligned} rS = rS_{\mathrm {max}} \left( \frac{S}{K_{\mathrm{s}}+S} \right) \times \prod _{i=1}^{n}{f_{\mathrm {Dsig}}(x_{\mathrm {i}})}. \end{aligned}$$

$K_{\mathrm{s}}$ is the half-saturation constant. The Monod-like term for the uptake rates is multiplied with the product of multiple double sigmoidal functions ($f_{\mathrm{Dsig}}$) of the state variables ($x_{\mathrm{i}}$), which describe changes of the cell metabolism [27, 41]:

4 $$\begin{aligned} f_\mathrm{Dsig}(x)= & {} \left( Y_{\mathrm{l}}+\frac{Y_{\mathrm{mid}}-Y_{l}}{1+e^{-K_{\mathrm{sl}}(x-X_{\mathrm{50,l}})}}\right) \nonumber \\&\times \left( 1+\frac{(Y_{\mathrm{h}}/Y_{\mathrm{mid}}-1)}{1+e^{-K_{\mathrm{sl}}(x-X_{\mathrm{50,\mathrm{h}}})}}\right) . \end{aligned}$$

The value of a state variable is described by *x*. $Y_{\mathrm{l}}$ is the value of $f_{\mathrm{Dsig}}$ at low *x*, and $Y_{\mathrm{h}}$ is the value at high *x*. $Y_{\mathrm{mid}}$ is the value between $X_{\mathrm{50,\mathrm{l}}}$ and $X_{\mathrm{50,\mathrm{h}}}$, which are location parameters of the low/high side of the function. $K_{\mathrm{sl}}$ determines the gradient of the slope [27]. The sigmoidal functions are also used to describe the influence of operating parameters on the activation and inactivation rates as well as yield coefficients. This structure enables the description of complex changes in multiple metabolic pathways and their intensity, e.g., for biomass formation, overflow metabolisms, biocatalysis, and complete oxidation under aerobic and anaerobic conditions. Moreover, the product of $f_{{\mathrm{Dsig}}}(x)$ is used to account for combined influences such as substrate/product inhibition and/or pH, DO, or temperature on the uptake rates. A parameterization strategy for the double sigmoidal functions is described in [41]. Each pathway is represented by the same, generalized stoichiometric function:

5 $$C_{x} H_{y} O_{z} + \nu _{1} O_{2} + \nu _{2} H_{g} O_{h} N_{i} \to \nu _{3} C_{a} H_{b} O_{c} N_{d} + \nu _{4} CO_{2} + \nu _{5} H_{2} O_{t} .$$

The stoichiometric coefficients $\nu _{i}$ were determined previously [42] and used according to:

6 $$\begin{aligned} Y_\mathrm {{i/S}} = \nu _{\mathrm {i}}\left( \frac{\mathrm{MW}_{i}}{\mathrm{MW}_{S}}\right) , \end{aligned}$$

where $\mathrm{MW}_{i}$ is the molecular weight for the state variable (e.g., biomass, $\mathrm{O}_2$, by-product) and ${\mathrm{MW}_{S}}$ for the substrate [27, 43]. The yield coefficients, describing the formation of a substance *i* based on the substrate S ($Y_{i/S}$) are used in the calculation of production and uptake rates, whereas rates of each pathway are summed up to total rates. The total rates $rc_{i}$ are then used in general mass balances for each component with the concentrations of components in the feed $c_{\mathrm {i,feed}}$ and their concentration in the bioreactor $c_{i}$:

7 $$\frac{{{\text{d}}c_{i} }}{{{\text{d}}t}} = \underbrace {{rc_{i}^{ + } \times Xv}}_{{{\text{production}}}} - \underbrace {{rc_{i}^{ - } \times Xv}}_{{{\text{uptake}}}} + \underbrace {{c_{{i,{\text{feed}}}} \times \frac{{F_{{c_{i} }} }}{V}}}_{{{\text{input}}}} - \underbrace {{c_{i} \times \frac{{F_{{V,{\text{in}}}} }}{V}}}_{{{\text{dilution}}}}.{\text{ }}$$

### Monte Carlo-based uncertainty quantification

To quantify the variability of the model simulations in the mDoE-toolbox, the model-parametric uncertainties are determined using Monte Carlo sampling with repeated parameter adaptations [19]. In brief, the determined standard deviation of each experimental data point was considered to be independent and normally distributed. For the initial values, the standard deviation was assumed to be 5%. The individual biomass compartments considered in the Six-compartment model (Section “Mathematical process model”) could not be experimentally determined and were presumed with a standard deviation of 10%. The standard deviations of the set pH value, temperature, DO, and feeding rates, as well as their concentrations, were defined to be 5% based on the typical standard deviations in bioprocesses (i.e., expert knowledge) [19, 44, 45]. The model-parametric uncertainty was determined based on the experimental uncertainty using multiple parameterization runs (Monte Carlo samples). Due to limited computational power, 116 adaptations were performed in case study S1 and 240 adaptations in case study S2. Model parameters were adapted by minimizing the weighted root-mean-square deviation *RMSD* [14, 19, 27, 46]. The *RMSD* is calculated from the squared difference between the measured value $y_{\mathrm{m}}$ and the simulated value $y_{\mathrm{s}}$, multiplied by a factor for weighting individual data points $k_{\mathrm{weighting}}$, and divided by the number of data points *n* in the data set:

8 $$\begin{aligned} {{RMSD = \sqrt{\sum _{i=1}^n \frac{(y_{s, i} - y_{m, i})^2}{n} \cdot k_{\mathrm{weighting}}}}}. \end{aligned}$$

Only high $c_{\mathrm{DCW}}>100~\mathrm{g~l}^{-1}$ were weighted by 0.5 and no weighting was used for other state variables. The individually adapted model parameters, their medians, 10% and 90% quantiles (ESM: Tables S1 and S2), as well as their distributions are shown in the ESM.

The simulations using the determined parameters were additionally evaluated using the coefficient of determination ($R^{2}$), which includes the differences between simulated $y_{s,i}$ and experimental data $y_{i}$ as well as the differences between experimental data and their mean ${\overline{y}}$ [1, 19, 47]:

9 $$\begin{aligned} R^{2} = 1-\frac{\sum _{i=1}^{n}(y_{i}-y_{\mathrm{s,i}})^2}{\sum _{i=1}^{n}(y_{i}-{\overline{y}})^2}. \end{aligned}$$

$R^2$ lies between minus infinity and one. If $R^2$ is one, the data points correspond precisely to the solution of the model. If $R^2$ is less than zero, the mean of the measured data points is closer to the mean result than the solution of the model [19].

### Planning of experimental design

The factor settings of the DoE designs were determined as described in the following: First, a large number of points ($>10^6$) were randomly distributed in the three-dimensional design space (i.e., three investigated factors). Then, clusters in these points were determined using the *k*-means algorithm [48, 49]. These clusters are partitioned into the *k* sets corresponding to the number of experiments in the DoE design. The resulting *k* cluster centers are the factor combinations (i.e., planned experiments) of the DoE design. Based on this algorithm and previous studies, a total of 29 experiments were planned, which were later individually simulated and evaluated using the mDoE-toolbox. The main advantage of this method, among other approaches, is the universal application to any number of investigated factors and experimental spaces of any shape [50].

### Monte Carlo-based simulation of planned experiments

Instead of performing each planned cultivation from the DoE design, they were first simulated (see Fig. 1) with the developed process model. Due to computational power, 30 Monte Carlo simulations (Section “Monte Carlo-based uncertainty quantification”) (C-eStlM, Germany) were performed for each planned experiment, so that the propagated uncertainty of the simulations was quantified. The model parameter values were drawn using Latin Hypercube Sampling using the R-Package “lhs” (V1.0.2) [51, 52]. The interval boundaries are determined by calculating the 10% and 90% quantiles using the Type R-7 method provided in R.

### Computational evaluation of experimental design

First, for each planned factor combination *i*, the average expected response (${\overline{r}}_{i}$) is calculated based on the Monte Carlo simulations. Furthermore, $\nu _{i}$ is calculated as the difference of the 10% and the 90% quantile, and is used as a measure of the expected process variability. In the mDoE-toolbox, the maximization of ${\overline{r}}_{i}$ is targeted with a simultaneously minimization of $\nu _{i}$. Therefore, for both ${\overline{r}}_{i}$ and $\nu _{i}$, individual desirability functions $d({\overline{r}}_{i} \mathrm{~ or}~\nu _{i}$) are calculated by rescaling between 0 and 1. $d({\overline{r}}_{{i}} \mathrm\,{\rm{or}}~\nu _{{i}})$ are based on the minimal response $L({\overline{r}} \mathrm\,{\rm{or}}~\nu )$ and the maximal response $U({\overline{r}} ~ \mathrm{or}~\nu )$ of all ${\overline{r}}_{{i}}$ and $\nu _{{i}}$ (vector including all *i* experiments donated as ${\overline{r}} \, \mathrm{or}~\nu )$. Therefore, the desirability function $d({\overline{r}}_{\mathrm{i}})$ is in the optimization range ($U({\overline{r}})$ – $L({\overline{r}})$). For the maximization of ${\overline{r}}_{\mathrm{i}}$, $d({\overline{r}}_{\mathrm{i}}$) is calculated as follows:

10 $$\begin{aligned} d({\overline{r}}_{\mathrm{i}})=\left( \frac{{\overline{r}}_{{i}} - L({\overline{r}})}{U({\overline{r}}) - L({\overline{r}})} \right) . \end{aligned}$$

For the minimization of $\nu _{i}$, *d*($\nu _{{i}}$) is inversely calculated, i.e., a high $\nu _{{i}}$ has a low *d*($\nu _{{i}}$) and *vice versa*:

11 $$\begin{aligned} d(\nu _{i}) = \left( \frac{\nu _{{i}} - U(\nu )}{L(\nu ) - U(\nu )} \right) . \end{aligned}$$

In the mDoE-toolbox, $d({\overline{r}}_{{i}})$ and $d(\nu _{i})$ are combined into one numerical value $D_{{i}}$ to quantify the average value and its variability of each planned experiment (see Section 1.1) into $D_{{i}}$ including weighting factors *w*:

12 $$\begin{aligned} {{ D_{{i}} = w_{\mathrm{1}} \times d({\overline{r}}_{{i}}) + w_{\mathrm{2}} \times d({\nu _{i}})}} \end{aligned}$$

for which

13 $$\begin{aligned} {{w_{\mathrm{1}} + w_{\mathrm{2}} = 1}}. \end{aligned}$$

By this approach, a risk-based evaluation of the planned designs is enabled and $w({\nu _{{i}}})$ reflects the percentage at which $\nu _{{i}}$ is considered. In this study, $w_{\mathrm{1}} =0.8$ and $w_{\mathrm{2}}= 0.2$. Contour and 3D plots were generated with Gnuplot 5.2.8.

## Results and discussion

The mDoE-toolbox software was tested on two optimization studies with *Saccharomyces cerevisiae* (S1 and S2, respectively). First, the aim was to maximize $c_{\mathrm{DCW}}$ after 48 h (S1) based on the experimental factors pH, as well as $F_{\mathrm{Glc}}$ and $F_{\mathrm{N}}$. Second, the biocatalytic conversion from EAA to E3HB was optimized (S2). E3HB should be maximized based on $F_{\mathrm{EAA}}$, $F_{\mathrm{Glc}}$ and $F_{\mathrm{N}}$. EAA shows inhibitory effects above a concentration of $c_{\mathrm{EAA}}= 0.5~\mathrm{g~l}^{-1}$ [53, 54]. In both processes, ethanol formation is crucial due to its inhibitory effect on cell growth and biocatalysis [55, 56]. In addition, *Saccharomyces cerevisiae* produces ethanol even under aerobic conditions if glucose concentration is above a certain limit. This phenomenon is known as the Crabtree effect and should be minimized to optimize growth and biocatalysis [57, 58].

### Monte Carlo-based uncertainty quantification

Model parameters and their distributions were determined using Monte Carlo sampling (see Fig. 1 box II), as explained in Section “Monte Carlo-based uncertainty quantification”. Therefore, three sets of experiments (see Table 1 and 2) were used. The first set consists of shaking flask experiments to adapt the parameters for the pH model. The second set consists of three historical fed-batch cultivations to model the growth of yeast, uptake rates, and production rates in relation to critical process parameters, e.g., glucose and ethanol concentration (both in Table 1). The last set was designed based on literature and was used to identify the parameters for the biocatalysis (Table 2).

#### Growth and metabolic model parameters (S1)

**Fig. 2:**
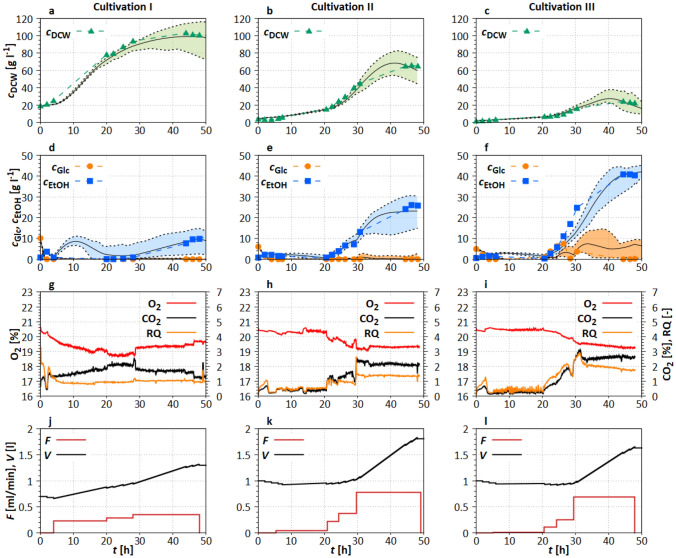
Comparison of experimental data to adapted model of three initial cultivations (see Table 1), **a**–**f** solid lines represent the mean of 116 Monte Carlo simulations (Section “Monte Carlo-based simulation of planned experiments”); dashed lines represent the 10% and 90% quantiles of the simulations; **g**–**i** online data of the off-gas measurement as well as the calculated respiratory quotient. **j**–**l** Calculated *V* and feeding rates (*F*). Experimental settings and the used reactor are shown in Section “Experiments for modeling”

The growth and metabolic model parameters targeted in case study S1 were adjusted using data of three fed-batch cultivations (see Table 1). These model parameters describe the uptake of glucose, ethanol, and nitrogen, the activation, inactivation, and mortality rates, as well as the general yield coefficients for glucose and ethanol. Furthermore, the parameters of the sigmoidal functions for the influence of glucose limitation and ethanol inhibition on the glucose and ethanol uptake as well as the biomass inactivation rate were identified (see ESM Section 3.1). The comparison of the experimental data with the Monte Carlo-based simulations including 10% and 90% quantiles are shown in Fig. 2a–f. Furthermore, gassing rates and experimental online data (Fig. 2g, h), and calculated total volume *V* and total feeding *F* (Fig. 2j–l) are shown.

Cultivations I and II were initial test cultivations aiming to achieve a high biomass density. For this purpose, different initial biomass concentrations were chosen. Cultivation III was performed with a feeding strategy which should lead to ethanol inhibition (Table 1).

**Cultivation I** In cultivation I, the biomass (Fig. 2a) increases from initially $c_{\mathrm{DCW}}= 18~ \mathrm{g~l}^{-1}$ to $\approx 100~\mathrm{g~l}^{-1}$ ($t=42~\mathrm{h}$). An initial $c_{\mathrm{DCW}}$ that high would not be used in “real” bioprocesses and was just utilized for the purpose of model parameter adaptation. The ethanol concentration was relatively low below $c_{\mathrm{EtOH}}=10~\mathrm{g~l}^{-1}$ throughout the experiment and glucose concentration was not measurable after $t=2~\mathrm{h}$. These results are reflected in the course of the respiratory quotient (Fig. 2b, RQ), which was constantly around one, indicating a low ethanol formation. Feeding rate was increased stepwise to $F= 0.35~\mathrm{ml~min}^{-1}$ (Fig. 2j).

**Cultivation II** In cultivation II, a biomass density of $c_{\mathrm{DCW}}=64~\mathrm{g~l}^{-1}$ was achieved at the end of the process and an ethanol concentration of $c_{\mathrm{EtOH}}=28~\mathrm{g~l}^{-1}$ was determined (Fig. 2d), leading to low growth inhibition. Glucose was directly consumed when fed, and therefore, the measured glucose concentrations were about $0~\mathrm{g~l}^{-1}$ after $t=10~\mathrm{h}$. The rather strong ethanol production after $t=30~\mathrm{h}$ is reflected in the RQ (Fig. 2e). Feeding (Fig. 2h) was higher than in cultivation I despite a lower initial biomass density.

**Cultivation III** As can be seen in Fig. 2c, only $c_{\mathrm{DCW}}=24~\mathrm{g~l}^{-1}$ was formed at $t=44~\mathrm{h}$. but over $c_{\mathrm{EtOH}}=40~\mathrm{g~l}^{-1}$ was produced during the same time period. This trend was also confirmed by the RQ (Fig. 2f), which was clearly above one from $t=20~\mathrm{h}$ onwards, indicating an increased $\mathrm{CO}_2$ formation during ethanol production. Feeding (Fig. 2i) was designed to induce ethanol inhibition (i.e., over-feeding) and was increased stepwise up to $F=~0.7~\mathrm{ml~min}^{-1}$ [59].

Overall, the model parameters could be adapted well to the process data with an $R^2$ above 0.85 (total for $c_{\mathrm{DCW}}$, $c_{\mathrm{Glc}}$, $c_{\mathrm{EtOH}}$) comparing the experimental data to the mean of the simulations for every experiment (Table 3).

**Table 3:** Total $R^2$ for average model parameters in Monte Carlo-based uncertainty quantification (S1—cultivation and S2—biocatalysis)

	Experiment	$R^{2}$
S1	Cultivation I	0.97
	Cultivation II	0.98
	Cultivation III	0.85
S2	Biocatalysis I	0.92
	Biocatalysis II	0.96

In addition, the modeling of high biomass densities and ethanol inhibition was adapted sufficiently. The width of the uncertainty band (10% and 90% quantiles) of the simulations (Fig. 2a–f) was narrow, indicating a reliable estimation of model parameters. All model parameters and their individual distribution are shown in ESM Figs. S3–S5.

#### pH-dependent model parameters (S1)

**Fig. 3:**
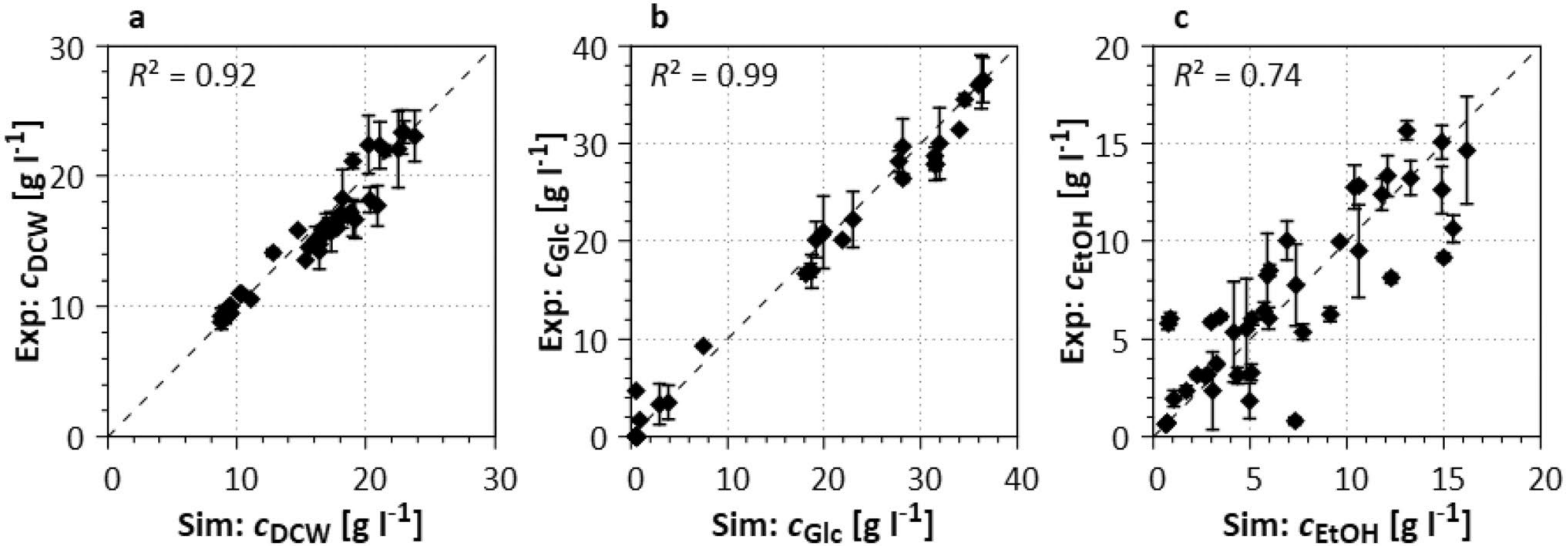
Comparison of experimental (Exp) data and simulated (Sim) data for the adaption of pH-related model parameters (see Section “Experiments for modeling”). Error bars show standard deviation of two parallel shaking flask experiments for each investigated pH (4, 5, 6, and 7, respectively). The quality of fit is represented by $R^2$ (optimal simulation *x* = *y*). For each pH, the individual growth curves are shown in the ESM Fig. S2. Experimental settings are specified in Section “Experiments for modeling”

As can be seen in Fig. 3, the influence of a wide pH range (4–7) on the growth and metabolism of *Saccharomyces cerevisiae* can be sufficiently reflected by the model with the adapted model parameters in case study S1. The concentrations of biomass and glucose can be well reflected with an $R^2$ above 0.92. The simulation of the ethanol concentration is sufficient ($R^2$ = 0.74).

For all cultivations (see ESM Fig. S2 for individual plots), cells grew up to approx. $c_{\mathrm{DCW}}= 20 - 25~\mathrm{g~l}^{-1}$ in the first hours including strong ethanol production. After glucose depletion, cell growth stagnated, and ethanol was taken up until the feed pulse at $t=24~\mathrm{h}$ was fed. After the feed pulse, the biomass density increased again, while glucose was consumed and ethanol produced. No strong growth inhibitions were seen for the different pH values investigated. The biomass densities at the end of the process were found to be slightly higher at pH 5 (compared to pH 4 and 6, respectively), and 10% higher than at pH 3. Furthermore, the glucose and ethanol consumption rates increased with increasing pH. The growth rates at pH 4 and pH 5 were equally the highest with $0.105 \pm 0.005$ $\mathrm{h}^{-1}$.

Using the experimental data, the model parameters related to the pH-dependent glucose metabolism and uptake, and the segregation of glucose into biomass and ethanol production were determined. These parameters were not used in the Monte Carlo parameter adaptation (Section “Monte Carlo-based uncertainty quantification”) to speed up the calculations of the parameterization algorithm and were kept constant thereafter.

#### Biocatalytic model part (S2)

**Fig. 4:**
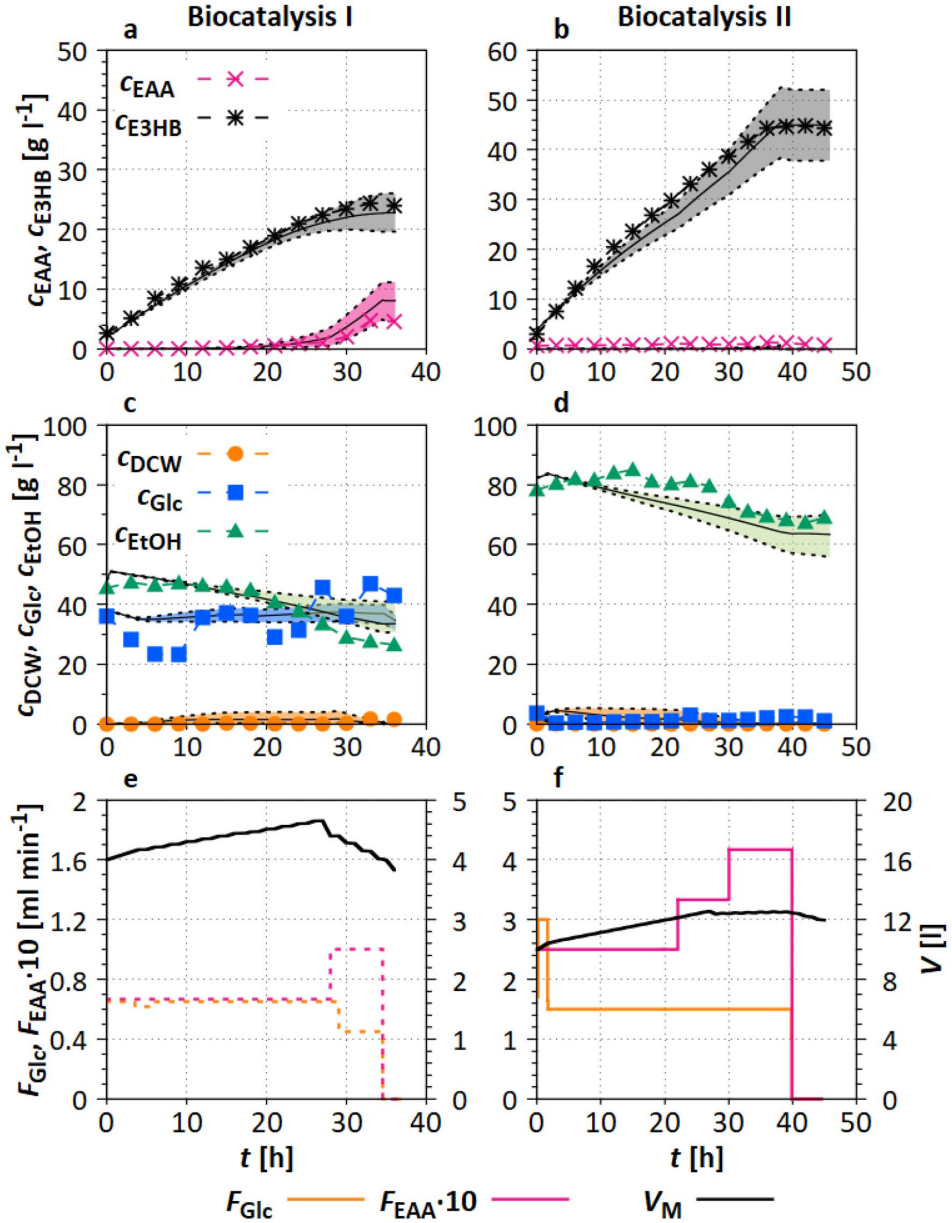
Model parameter adaptation for biocatalysis (see Table 2). **a**–**d** solid line represents the mean of 116 Monte Carlo simulations (Section “Monte Carlo-based simulation of planned experiments”), dashed lines represent the 10% and 90% quantiles of the simulations; **e**, **f** calculated *V* and *F*. Experimental settings and used reactors are specified in Section “S2: optimization of fed-batch strategy for biocatalysis”

The biocatalysis model used in S2 was adapted on data of two experiments, based on the literature [53, 54]. The focus was on those model parameters, characterizing the biocatalysis, and therefore partly differs from the previously chosen parameters in case study S1. The new parameters are listed in ESM Section 3.2 and describe the uptake of EAA, glucose, ethanol and nitrogen, the activation, inactivation, and mortality rate, as well as the general yield coefficients for EAA, glucose, and ethanol. Furthermore, the parameters of the sigmoidal functions quantifying the influence of ethanol inhibition, glucose limitation on glucose uptake, and EAA inhibition on the biomass inactivation rate were adapted to the experimental data. In Fig. 4, the comparison between experimental and simulation data of the biocatalysis parameterization experiments is shown.

**Biocatalysis I** The first experiment aimed at the production of E3HB with $c_{\mathrm{DCW}}=45~\mathrm{g~l}^{-1}$. The feed and initial concentrations of the parameterization experiments are listed in Table 2. In the first experiment, biomass (Fig. 4a) decreased to $c_{\mathrm{DCW}}=25~\mathrm{g~l}^{-1}$ ($t=26~\mathrm{h}$), first due to dilution by feeding and towards the end due to a higher mortality rate induced by toxically high concentrations of EAA. Ethanol concentration (Fig. 4c) rose from $30~\mathrm{g~l}^{-1}$ to over $40~\mathrm{g~l}^{-1}$ at $t=26~\mathrm{h}$. Feeding was designed to increase $c_{\mathrm{EAA}}$ (Fig. 4e). Therefore, a constant $F_{\mathrm{Glc}}= 0.65~\mathrm{ml~min}^{-1}$ and a constant $F_{\mathrm{EAA}}=0.067~\mathrm{ml~min}^{-1}$ were set during the first 28 h. Then, $F_{\mathrm{Glc}}$ was reduced and $F_{\mathrm{EAA}}$ was increased to identify potential EAA inhibition. Thus, $c_{\mathrm{EAA}}$ rose to over 4 $\mathrm{g~l}^{-1}$, and the resulting inhibition is reflected in rising $c_{\mathrm{Glc}}$, despite the reduced glucose feed. E3HB constantly increased up to $c_{\mathrm{E3HB}}=24~\mathrm{g~l}^{-1}$ ($t=32~\mathrm{h}$) and then stopped increasing due to the EAA and potential ethanol inhibition.

**Biocatalysis II** In the second experiment, a higher initial biomass concentration of $c_{\mathrm{DCW}}=80~\mathrm{g~l}^{-1}$ was used, and ethanol and glucose concentrations were kept below 0.1 $\mathrm{g~l}^{-1}$ during the whole experiment (Fig. 4b, d). As in Cultivation I (S1), this high $c_{\mathrm{DCW}}$ was used for model parameter adaptation. After 48 h, $c_{\mathrm{E3HB}}= 44~\mathrm{g~l}^{-1}$ was reached, whereas $c_{\mathrm{EAA}}$ was constantly low and the biomass density decreased due to dilution. Constant glucose ($F_{\mathrm{Glc}}=3~\mathrm{ml~min}^{-1}$) and EAA feeding rates ($F_{\mathrm{EAA}}=0.05~\mathrm{ml~min}^{-1}$) were set. $F_{\mathrm{EAA}}$ was increased to $0.085~\mathrm{ml~min}^{-1}$ in two steps.

Overall, the parameterized model parameters reflect the kinetics of biocatalysis satisfactorily. The calculated $R^2$ are shown Table 3 and are higher than 0.92 for both simulations for the experimental data compared to the mean simulation (summarized for $c_{\mathrm{E3HB}}$, $c_{\mathrm{EAA}}$, $c_{\mathrm{DCW}}$, $c_{\mathrm{EtOH}}$).

The biocatalytic metabolite concentrations are well reproduced by the model for both experiments. The 10% and 90% quantiles of the simulations (Fig. 4a–d) are small. The model parameters and their individual distributions are shown in ESM Figs. S6–S9.

### Optimization of fed-batch process with mDoE-toolbox (S1)

The factors (pH, $F_{\mathrm{Glc}}$, $F_{\mathrm{N}}$, respectively) have been selected based on literature and experience in the cultivation of *Saccharomyces cerevisiae*. pH is described to have a strong influence on viability and growth rate [60]. Glucose is essential for cell growth, but high glucose feeding rates lead to ethanol formation due to the Crabtree effect and possibly ethanol inhibition [58–59]. A nitrogen source is essential for cell growth, but feeding needs a tight control to avoid dilution and to enable steady growth [61].

#### mDoE-toolbox (S1)

**Planning of experimental design and Monte Carlo simulations** Using the mDoE-toolbox explained in Sect. 1.1, rather widely distributed initial boundary values are defined first for the planning of the experimental design (Fig. 1, box III) [1]. Therefore, start of feeding was at $t= 1 \mathrm{~h}$. The initial boundaries for $F_{\mathrm{Glc}}$ were set between $0.1 \le \textit{F}_{\mathrm{Glc}} \le 1~\mathrm{ml~min}^{-1}$, and $\textit{F}_{\mathrm{N}}$ between $0.05 \le \textit{F}_{\mathrm{N}} \le 0.6~\mathrm{ml~min}^{-1}$. pH was varied between 3 and 7. Using the mDoE-toolbox, a total of 29 design points (i.e., planned experiments) were distributed in the three-dimensional design space, determined by the previously defined boundaries (see Section “Planning of experimental design”). For each of the planned experiments, Monte Carlo simulations (Fig. 1, box IV) were performed, as explained in Section “Monte Carlo-based simulation of planned experiments”.

**Fig. 5:**
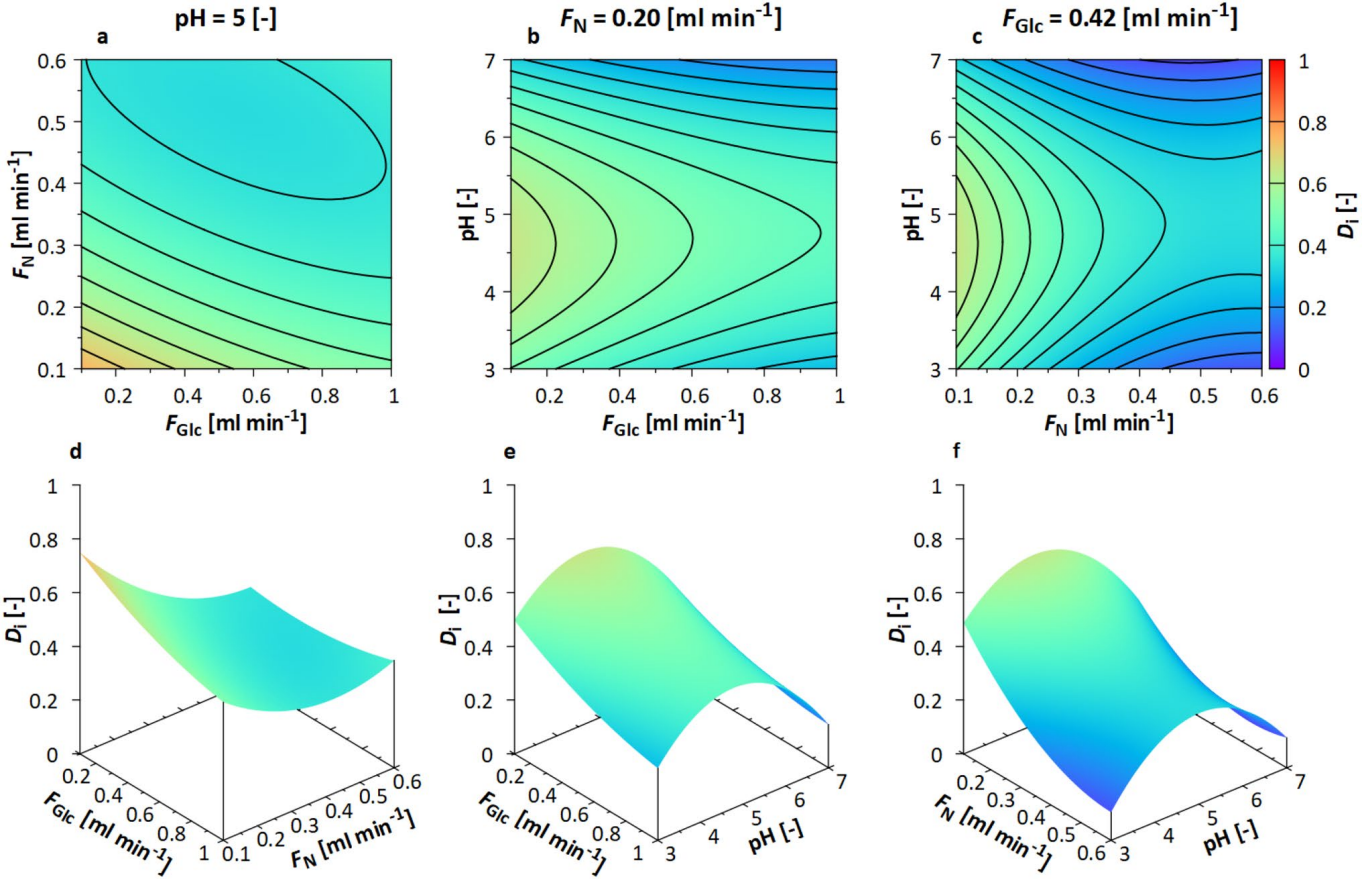
Contour and 3D plot for the response surfaces based on $D_{{i}}$ for the optimization of pH, $F_{\mathrm{Glc}}$, and $F_{\mathrm{N}}$ in S1. The responses were calculated with Monte Carlo simulations in the mDoE-toolbox, as explained in Section 1.1. **a**–**c** Graphs are adjusted to $\mathrm{pH} = 5$, $F_{\mathrm{N}} = 0.20 \, \mathrm{ml} \, \mathrm{min}^{-1}$ and $F_{\mathrm{Glc}} = 0.42 \, \mathrm{ml} \, \mathrm{min}^{-1}$, respectively, lines show differences of 0.05; **d**–**f** 3D representation of RS curves. The individual quadratic RS equations are shown in ESM Table S1

**Computational evaluation of experimental design** From the Monte Carlo simulations, ${\overline{r}}_{\mathrm{i}}$ and $\nu _{i}$ were calculated for the maximum $c_{\mathrm{DCW}}$ for each experimental setting *i*, which were further used to derive the desirability $D_{\mathrm{i}}$ (Fig. 1, box V). In Fig. 5, the desirability functions are plotted for pH = 5.0, $F_{\mathrm{N}}= 0.20~\mathrm{ml~min}^{-1}$, and $F_{\mathrm{Glc}}= 0.42~\mathrm{ml~min}^{-1}$, for which the highest $D_{\mathrm{i}}$ was calculated. Figure 5 shows the contour and 3D plots at these process conditions.

$D_{{i}}$ is high for flow rates of $F_{\mathrm{Glc}}~<~0.5~\mathrm{ml~min}^{-1}$ and $F_{\mathrm{N}}~<~0.3~\mathrm{~ml~min}^{-1}$ at pH = 5.0 (Fig. 5a). The influence of the pH value (Fig. 5b) on the desirability function is low, and a pH between 4 and 6 provides the best results. These results were additionally confirmed with the RSM in Fig. 5c and were low $F_{\mathrm{N}}~<~0.3~\mathrm{~ml~min}^{-1}$, and a pH between 4 and 6 shows again the best results. Using the mDoE-toolbox, the experimental space was computationally simulated and evaluated. For the factors investigated, the resulting RS plots could hardly be predicted on experience solely. The in silico calculation and computational evaluation of the planned experimental design offer a knowledge-driven approach, which is the major advantage of the mDoE-toolbox.

**mDoE-suggested experimental settings** Based on the evaluation of the experimental space, further experiments were recommended (see Sect. 2.8) from the toolbox. To statistically validate the recommended factor settings, four experiments located in the high $D_{{i}}$ region were chosen, which are listed in Table 4.

**Table 4:** Factor combination of the four yeast cultivation experiments determined with the usage of the mDoE-toolbox. $F_{\mathrm{Glc}}$ and $F_{\mathrm{N}}$ are the final maximum feed rates of the linearly rising feed at the end of the experiments

Exp.	$F_{\mathrm{Glc}}$	$F_{\mathrm{N}}$	pH
	($\mathrm{ml~min}^{-1}$)	($\mathrm{ml~min}^{-1}$)	(–)
#1	0.28	0.12	4
#2	0.28	0.12	6
#3	0.41	0.18	5
#4	0.54	0.25	5

#### Performed experiments

**Fig. 6:**
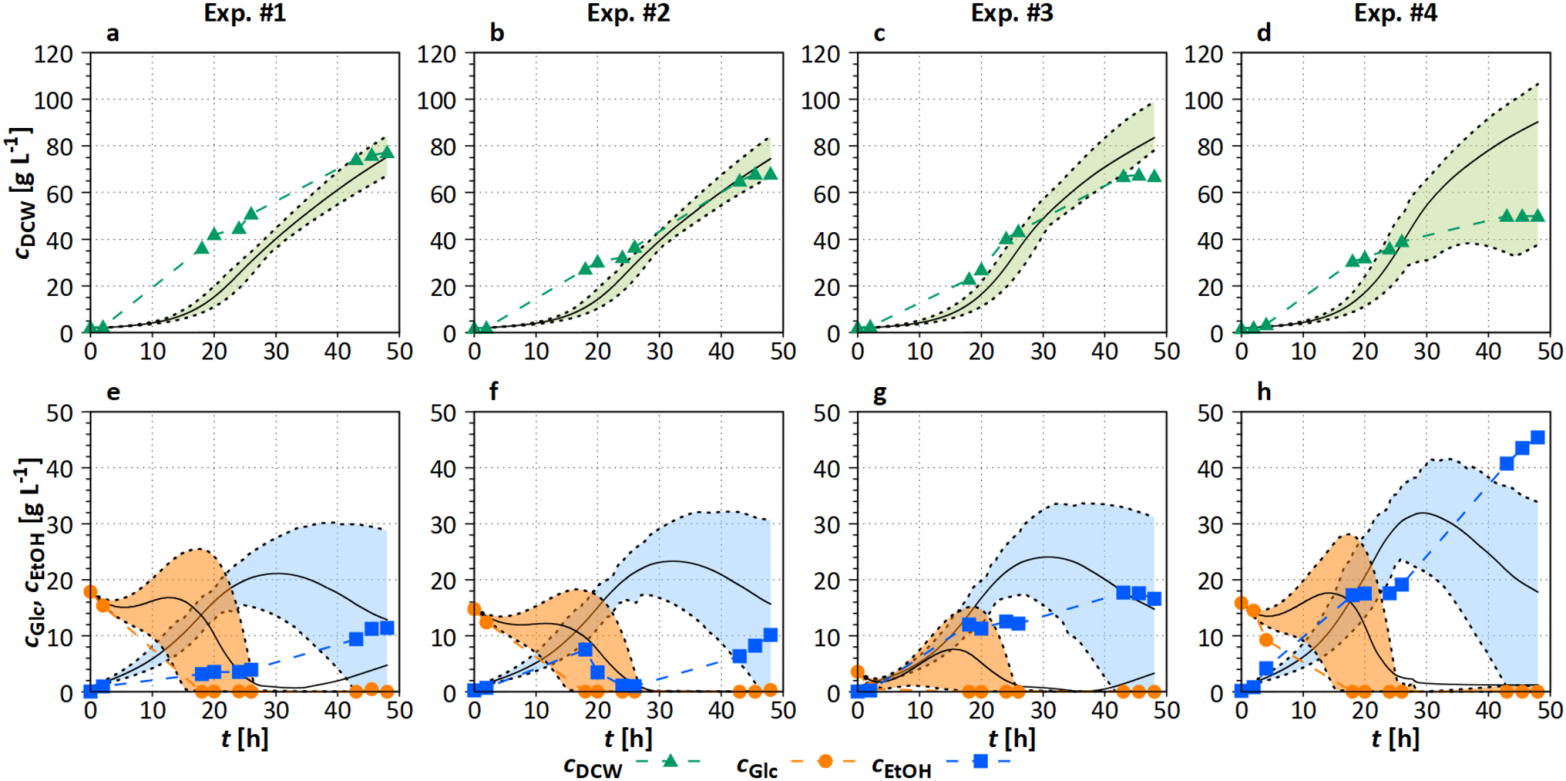
Experimental data of the four performed cultivations compared to the simulated data from the mDoE-toolbox for $c_{\mathrm{DCW}}$, $c_{\mathrm{Glc}}$, and $c_{\mathrm{EtOH}}$. The solid line is the mean of 30 simulations (Section “Monte Carlo-based simulation of planned experiments”); dashed line represents the 10% and 90% quantiles of the simulations, and online off-gas data and individual feeding profiles can be found in ESM Fig. S10. Experimental settings and the used reactor are specified in Section “Recommended experiments from mDoE-toolbox”

The four recommended experiments were performed (see Section “Recommended experiments from mDoE-toolbox”). The comparison of the experimental data to the model simulations, including the parametric 10% and 90% uncertainty-based prediction bands, is depicted in Figure 6.

**Biomass** In all four cultivations, cells grew until maximum biomass densities between $c_{\mathrm{DCW}}= 45~\mathrm{g~l}^{-1}$ (Fig. 6d) and $c_{\mathrm{DCW}}= 80~\mathrm{g~l}^{-1}$ (Fig. 6a). Growth was predicted sufficiently for all cultivations and only partly underestimated between $t= 18~\mathrm{and}~24~\mathrm{h}$ in cultivation #4 (Fig. 6c). The width of the uncertainty band (10% and 90% quantiles) was relatively narrow for cultivation #1–#3 (Fig. 6a–c), which reflects that the variability of these experimental settings are predicted to be low. Broad uncertainty bands indicating a high variability of the experimental settings were predicted for cultivation #4 (Fig. 6d), due to high $c_{\mathrm{EtOH}}$ near inhibitory conditions.

**Glucose and ethanol** Glucose was consumed during the cultivations and was constantly very low (i.e., fully consumed) after $t\approx 20~\mathrm{h}$. In cultivations #1 and #2, $c_{\mathrm{EtOH}}$ increased only at the end of the cultivation in relatively low amounts below $c_{\mathrm{EtOH}}=12~\mathrm{g~l}^{-1}$, for which no growth inhibition was seen. In cultures #3 and #4 (Fig. 6g, h), ethanol was not consumed and produced up to a maximum inhibitory concentration of $c_{\mathrm{EtOH}}=45.5~\pm ~1.8~\mathrm{g~l}^{-1}$ in cultivation #4 (Fig. 6h) [59, 61].

Overall, $c_{\mathrm{DCW}}= 80~\mathrm{g~l}^{-1}$ was achieved (cultivation #1) after the application of the mDoE-toolbox in a study with three influencing factors ($F_{\mathrm{Glc}}$, $F_{\mathrm{N}}$, pH). This reflects an improvement of $\approx 30\%$ in relation to the cultivation II (Fig. 2 b) with similar initial conditions. Simultaneously, $c_{\mathrm{EtOH}}$ at $t= 48~\mathrm{h}$ could be reduced by 50% compared to cultivation II (Fig. 2 e), resulting in higher substrate usage and a safer process operation point due to less possibility of inhibition.

The investigated factors are difficult to asses in traditional DoE studies due to their dynamic nature, i.e., the feeding rate itself changes during the process. A model-based approach strongly supports the evaluation of such dynamically changing factors.

### Optimization of biocatalytic conversion of EAA to E3HB (S2)

In case study S2, the feeding rates $F_{\mathrm{EAA}}$, $F_{\mathrm{Glc}}$, and $F_{\mathrm{N}}$ were manipulated to optimize the biocatalytic conversion of EAA to E3HB. The optimization objective is defined as the maximization of the biocatalytic product concentration ($c_{\mathrm{E3HB}}$). Among the other factors, $F_{\mathrm{EAA}}$ is very critical, because $c_{\mathrm{EAA}}$ has to be kept below $0.5~\mathrm{g~l}^{-1}$ to avoid inhibition [53].

#### mDoE-toolbox (S2)

**Planning of experimental design and Monte Carlo simulations** The boundaries for $F_{\mathrm{EAA}}$ were $0 \le \textit{F}_{\mathrm{EAA}} \le 0.04~\mathrm{ml~min}^{-1}$, based on literature to avoid inhibition through high EAA concentrations [53, 54]. $F_{\mathrm{Glc}}$ and $F_{\mathrm{N}}$ were defined to meet the demands the maintenance metabolism and the amount required for biocatalysis to be $0 \le \textit{F}_{\mathrm{Glc}} \le 0.2~\mathrm{ml~min}^{-1}$ and $0 \le \textit{F}_{\mathrm{N}} \le 0.03~\mathrm{ml~min}^{-1}$. The constitution of the feeds can be found in Table 2. For each simulated and performed biocatalysis, $c_{\mathrm{DCW}}=30~\mathrm{g~l}^{-1}$ was directly inoculated with no prior cultivation. The same design of the experiments as in S1 was applied (see Fig. 1 box III) and 29 experiments were planned initially using the mDoE-toolbox. For each planned experiment, Monte Carlo simulations were performed (see Fig. 1, box IV).

**Fig. 7:**
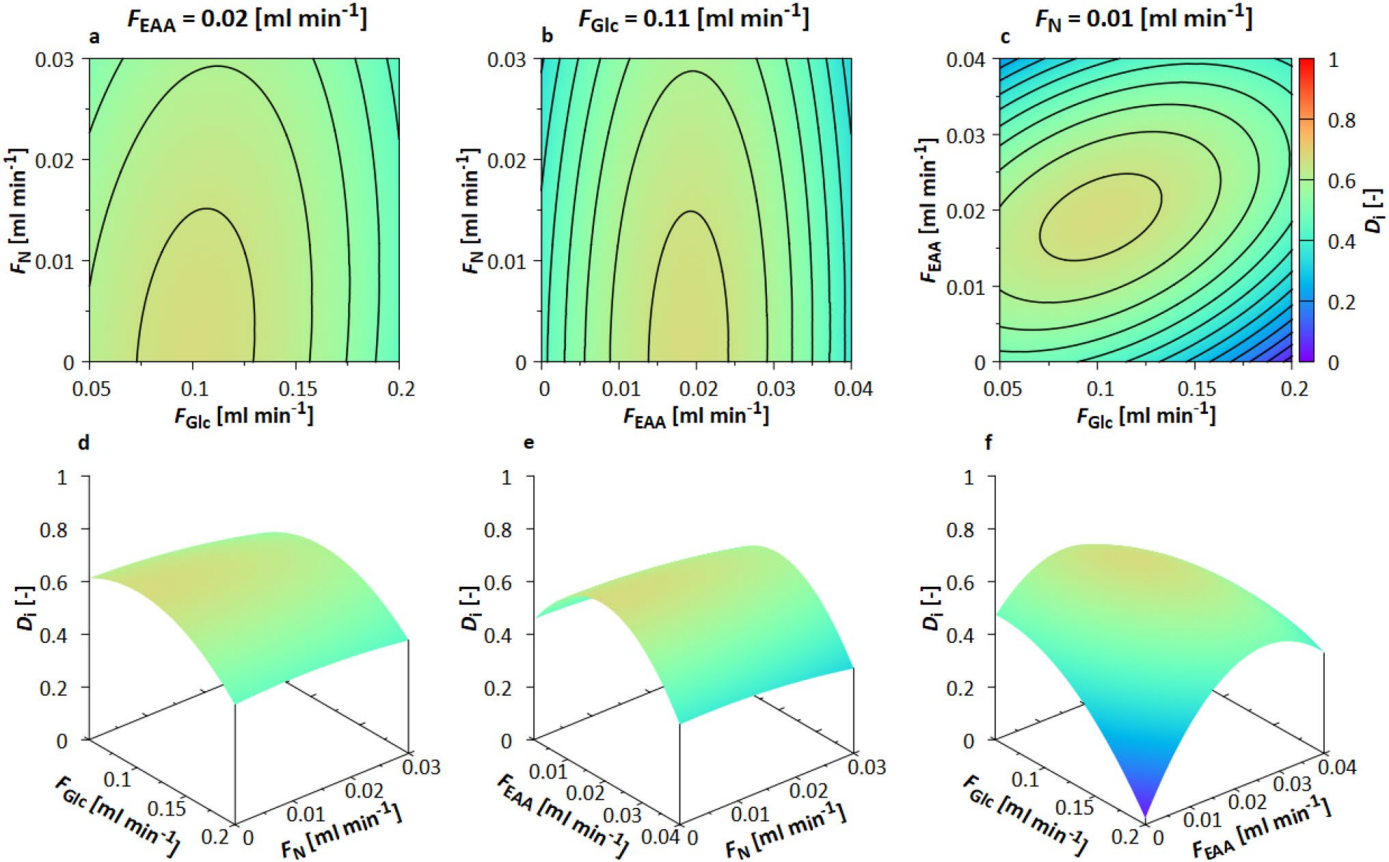
Contour and 3D plot for the response surfaces based on $D_{{i}}$ for $F_{\mathrm{EAA}}$, $F_{\mathrm{Glc}}$, and $F_{\mathrm{N}}$ in case study S2. The responses were calculated with Monte Carlo simulations in the mDoE-toolbox, as explained in Section 1.1. **a**–**c** Graphs are adjusted to $F_{\mathrm{EAA}} = 0.02 \, \mathrm{ml} \, \mathrm{min}^{-1}$, $F_{\mathrm{Glc}} = 0.11 \mathrm \, \mathrm{ml} \, \mathrm{min}^{-1}$ and $F_{\mathrm{N}} = 0.01 \, \mathrm{ml} \, \mathrm{min}^{-1}$ respectively, lines show differences of 0.05; **d**–**f** 3D representation of RS curves. The individual quadratic RS equations are shown in ESM Table S1

**Computational evaluation of experimental design** $D_{{i}}$ was calculated and the response surface was predicted (Fig. 1 box V). As can be seen in Fig. 7, a maximum was determined at ${F}_{\mathrm{EAA}}=0.02~\mathrm{ml~min}^{-1}$, ${F}_{\mathrm{Glc}}= 0.11~\mathrm{ml~min}^{-1}$, and ${F}_{\mathrm{N}}=0.01~\mathrm{ml~min}^{-1}$.

Excessive EAA feeding is predicted to inhibit and deactivate biocatalysis. A high $D_{{i}}$ is achieved only with a low ${F}_{\mathrm{EAA}}$, as can be seen from the shape of the 3D plots (Fig. 7d–f). Too small values of ${F}_{\mathrm{Glc}}$ lead to low ${c}_{\mathrm{Glc}}$ limiting the biocatalysis in the simulations. Too high ${F}_{\mathrm{Glc}}$ resulting in an increased ${c}_{\mathrm{Glc}}$, which leads to ethanol formation due to the Crabtree effect. In addition, high feeding rates (${F}_{\mathrm{Glc}}$ and ${F}_{\mathrm{N}}$) always result in dilution. The impact of ${F}_{\mathrm{N}}$ on achieving high $c_{\mathrm{E3HB}}$ is rather low. Therefore, a small ${F}_{\mathrm{N}}$ is desirable.

**mDoE-suggested experimental settings** Based on the Monte Carlo simulations and the computational evaluation of the experimental space, experiments located in the high $D_{\mathrm{i}}$ regions were identified and four of them were chosen. The experimental settings are listed in Table 5.

**Table 5:** Factor combination of the four recommended biocatalytic experiments determined with the mDoE-toolbox

Exp.	$F_{\mathrm{EAA}}$	$F_{\mathrm{Glc}}$	$F_{\mathrm{N}}$
	($\mathrm{ml~min}^{-1}$)	($\mathrm{ml~min}^{-1}$)	($\mathrm{ml~min}^{-1}$)
#1	0.024	0.09	0.010
#2	0.018	0.12	0.010
#3	0.014	0.06	0.020
#4	0.010	0.09	0.002

#### Performed experiments

**Fig. 8:**
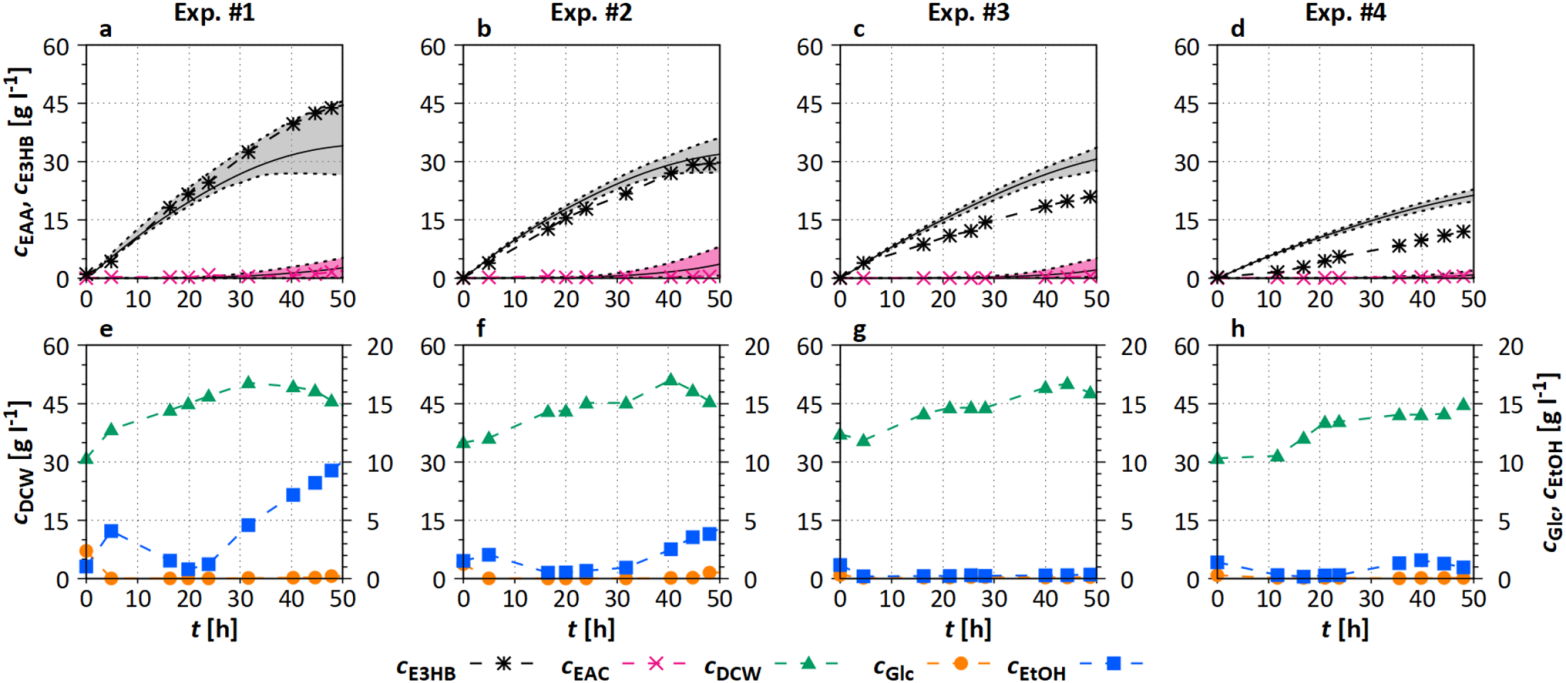
Experimental data of the four performed cultivations compared to the simulated data from the mDoE-toolbox for $c_{\mathrm{EAA}}$, $c_{\mathrm{E3HB}}$, and $c_{\mathrm{Glc}}$. The solid line is the mean of 30 simulations (Section “Monte Carlo-based simulation of planned experiments”); dashed line represents the 10% and 90% quantiles of the simulations. Experimental settings and the used reactor are specified in Section “Recommended experiments from mDoE-toolbox”

As can be seen in Fig. 8, final E3HB concentrations between $c_{\mathrm{E3HB}}= 10-50~\mathrm{g~l}^{-1}$ were reached in the four recommended experiments.

**EAA and E3HB** Even if the variation in ${F}_{\mathrm{EAA}}$ in the four experiments was relatively low ($0.10 \le \textit{F}_{\mathrm{EAA}} \le 0.24~\mathrm{ml~min}^{-1}$), the maximum ${c}_{\mathrm{E3HB}}$ decreased strongly with decreasing ${F}_{\mathrm{EAA}}$ from cultivations #1-#4. EAA was constantly consumed during the bioprocesses and reached a maximum of $c_{\mathrm{EAA}}=2~\mathrm{g~l}^{-1}$ in experiment #1 (Fig. 8a). The width of the 10% and 90% quantiles of the $c_{\mathrm{EAA}}$ simulations increases for higher $F_{\mathrm{EAA}}$, due to an increasing probability of EAA inhibition.

In cultivations #3 and #4 (Fig. 8c, d), $c_{\mathrm{E3HB}}$ is lower than the prediction. Since no EAA was detectable, a higher by-product formation rate might have occurred in the experiments with lower product concentrations (Fig. 8c, d).

**Biomass, glucose, and ethanol** Despite a low ${F}_{\mathrm{N}}$, biomass density increased by at least 15 $\mathrm{g~l}^{-1}$ in every experiment (Fig. 8e–h). This was consistent with $c_{\mathrm{Glc}}$ and $c_{\mathrm{EtOH}}$, which were below 5 $\mathrm{g~l}^{-1}$ for cultivations #2-#4. In cultivation #1, increasing $c_{\mathrm{EAA}}$ leads to inhibition and reduction of the metabolic activity, resulting in a lower glucose consumption and an increasing glucose concentration up to $c_{\mathrm{Glc}}=1~\mathrm{g~l}^{-1}$. This induces ethanol formation reaching $c_{\mathrm{EtOH}}=10~\mathrm{g~l}^{-1}$ ($t= 48~\mathrm{h}$, Fig. 8e).

It could be shown that even the feeding strategy for a biocatalytic process with complex reaction mechanisms could be designed and optimized with the application of the mDoE-toolbox. By calculating an optimal feeding profile for EAA, glucose, and nitrogen source, $c_{\mathrm{E3HB}}=44~\mathrm{g~l}^{-1}$ (Exp. #1) was achieved. This is an improvement of 80% in comparison to the experiment Biocatalysis I (Fig. 4a), with similar initial conditions. The same E3HB concentration was reached with a 60% lower initial $c_{\mathrm{DCW}}$, when compared to the high cell density experiment (Biocatalysis II, Fig. 4b) and 10% compared to the literature [53, 54]. Furthermore, the application of the mDoE-toolbox reduced the initial experimental space by over 90% (compare Table 4 with Fig. 5). Only four recommended experiments had to be performed to find improved operating conditions, resulting in 80% higher product concentrations. The reactor volume was 80% smaller compared to Biocatalysis I, which may have had an additional influence on the improved product concentration. However, the transferability of process understanding obtained using mathematical process models was recently shown to be transferable between different scales [19, 37].

## Conclusion

In this study, the mDoE-toolbox was introduced to enable a more knowledge-driven experimental design, and to strongly reduce the number of experiments during bioprocess development and optimization. The application of the toolbox was shown for two different case studies with *Saccharomyces cerevisiae*. In case study S1, a fed-batch process was optimized to maximize the final biomass density depending on the factors pH, and linearly rising substrate feeding rates. Just four experiments were needed to achieve a 30% increase of the final biomass density compared to Cultivation II, instead of 29 initially planned experiments, which would have been performed in the fully experimental evaluation of the DoE. In case study S2, the biocatalytic production of E3HB was optimized based on constant substrate feeding rates. Just four experiments were needed instead of 29 initially planned experiments. An improvement of 80% in the final E3HB concentration was experimentally achieved compared to the experiment Biocatalysis I with similar initial conditions. Although this reaction is well known, an improvement of about 10% of $c_{\mathrm{E3HB}}$ was achieved compared to the literature [53, 54]. In addition, this result was obtained in less than half the cultivation time [54]. In both processes, the optimization of rather difficult to assess factors, such as timely changing feeding rates (S1) or feeding of inhibitory components (S2), was possible through modeling.

In summary, the usage of the mDoE-toolbox enables optimization studies with dynamic factors in statistics-based biotechnology research employing a reduced number of experiments. Further research will focus on online model parameter adaptation and the consequent online re-design of experiments to increase the obtained process understanding further.

## Supplementary Information

Below is the link to the electronic supplementary material.

Supplementary file1 (PDF 1202 KB)

## Abbreviations

DCW: Dry cell weight
DO: Dissolved oxygen
DoE: Design of experiments
EAA: Ethyl acetoacetate
EtOH: Ethanol
E3HB: (*S*)-Ethyl-3-hydroxybutyrate
Glc: Glucose
mDoE: Model-assisted design of experiments
Pep: Peptone
*RMSD*: Weighted mean squared deviation
RS: Response surface
RSM: Response surface method
RQ: Respiratory quotient
YE: Yeast extract

## List of symbols

$c_{\rm k}$: Concentration of component k in the bioreactor (${\mathrm{g~l}}^{-1}$)
$c_{{{\text{k,feed}}}}$: Concentration of component k in the feed (${\mathrm{g~l}}^{-1}$)
$${{d}}$$: Desirability function (–)
$D_{i}$: Combined desirability function (–)
*F*: Total feeding rate ($\mathrm{ml~min}^{-1}$)
$F_{c_{\mathrm{k}}}$: Feeding rate of component k ($\mathrm{ml~min}^{-1}$)
$F_{\mathrm{k}}$: Feeding rate of component k ($\mathrm{ml~min}^{-1}$)
$F_{\mathrm{V, in}}$: Total volumetric feed rate (${\mathrm{g~l}}^{-1} \mathrm{h}^{-1}$)
*i* (index): Recommended factor combination (–)
*k* (index): EAA, Glc, N, EtOH, E3HB (–)
$k_{\mathrm{weighting}}$: Weighting factor in RMSD (–)
$$K_{\mathrm{s}}$$: Saturation constant ($${\mathrm{g~l}}^{-1}$$)
$$K_{\mathrm{sl}}$$: Gradient slope (–)
${{L}}$: Lowest value/lower limit (–)
$\mathrm{MW}_{\mathrm{k}}$: Molecular weight of the state variable *k* ($\mathrm{g~mol}^{-1}$)
$\mathrm{MW}_{\mathrm{S}}$: Molecular weight of the substrate S ($\mathrm{g~mol}^{-1}$)
*n*: Number of data points (–)
$r_{c_{\mathrm{k}}}$: Total uptake/consumption rate ($\mathrm{h}^{-1}$)
${\bar{r}}_{{i}}$: Average value of response i (–)
$r_{{S}}$: Substrate uptake rate (${\mathrm{g~l}}^{-1} \mathrm{h}^{-1}$)
$R^2$: Coefficient of determination (–)
$$S_{\mathrm{BC}}$$: Biocatalysis educt ($$\mathrm{g~l}^{-1}$$)
$$S_{\mathrm{C}}$$: Carbon substrate ($$\mathrm{g~l}^{-1}$$)
$$S_{\mathrm{N}}$$: Nitrogen substrate ($$\mathrm{g~l}^{-1}$$)
*t*: Time (h)
${{U}}$: Highest value/upper limit (–)
*V*: Working volume of the bioreactor (l)
${{w}}$: Weighting factor in $D_{{i}}$ (–)
Xd: Dead biomass ($\mathrm{g~l}^{-1}$)
Xi: Inactive biomass ($\mathrm{g~l}^{-1}$)
Xp: Product forming biomass ($\mathrm{g~l}^{-1}$)
Xpri: Autocatalytically active biomass ($\mathrm{g~l}^{-1}$)
Xs: Structurally active biomass ($\mathrm{g~l}^{-1}$)
Xsi: Structurally inactive biomass ($\mathrm{g~l}^{-1}$)
$$X_{\mathrm{v}}$$: Viable biomass ($$\mathrm{g~l}^{-1}$$)
$X_{\mathrm{50,l}}$: Low location parameter of x (–)
$X_{\mathrm{50,h}}$: High location parameter of x (–)
$$y_{i}$$: Measured value (–)
$\bar{y_{\mathrm{i}}}$: Mean of measured data (–)
$$y_{\mathrm{s}}$$: Simulated value (–)
$$Y_{\mathrm{h}}$$: *y* at high *x* (–)
$$Y_{i/S}$$: Yield coefficient (–)
$$Y_{\mathrm{l}}$$: *y* at low *x* (–)
$$Y_{\mathrm{mid}}$$: *y* at medium *x* (–)
$\nu _{i}$ in model: Stoichiometric coefficient (–)
$\nu _{i}$ in MC simulation: Predicted variability (–)
