# Supplementary material for: Model-assisted DoE software: optimization of growth and biocatalysis in Saccharomyces cerevisiae bioprocesses
Source: Bioprocess Biosyst Eng. 2021 Jan 20;44(4):683–700. doi: 10.1007/s00449-020-02478-3 (PMC7997827; doi:10.1007/s00449-020-02478-3)
Supplement: Supplementary file 1 — Supplementary file1 (PDF 1202 KB) [file 449_2020_2478_MOESM1_ESM.pdf]

Electronic Supplementary Material to: Model-assisted DoE software: Optimization of growth and biocatalysis in *Saccharomyces cerevisiae* bioprocesses

André Moser[1], Kim B. Kuchemüller[2], Sahar Deppe[3], Tanja Hernández Rodríguez[3], Björn Frahm[3], Ralf Pörtner[2], Volker C. Hass[1], Johannes Möller[2,4]

October 2, 2020

1 Mathematical process model

The structure of the utilized process model (Six Compartment model) is shown in Figure S1.

The following compartments are considered: An autocatalytically active biomass (Xpri), a product forming (Xp), a biocatalitically inactive (Xi), a structurally active (Xs) and inactive (Xsi) and a dead biomass (Xd) compartment. Biomass synthesis is based on a carbon (SC), amino acids (SAA), a nitrogen substrate (SN) and on a carbon product (PC) under diauxic growth. The biocatalysis is modeled based on an educt (SBC) (not shown). Furthermore, components of the biomass compartments and connections between the compartments are shown.

2 Adaption of model parameters

The individual plots used for the adaption of the pH-model related parameters are shown in Figure S2.

[1]Furtwangen University of Applied Sciences, Faculty of Medical and Life Sciences

[2]Hamburg University of Technology, Institute of Bioprocess and Biosystems Engineering

[3]Ostwestfalen-Lippe University of Applied Sciences and Arts, Biotechnology and Bioengineering

[4](corresponding author), e-mail: johannes.moeller@tuhh.de

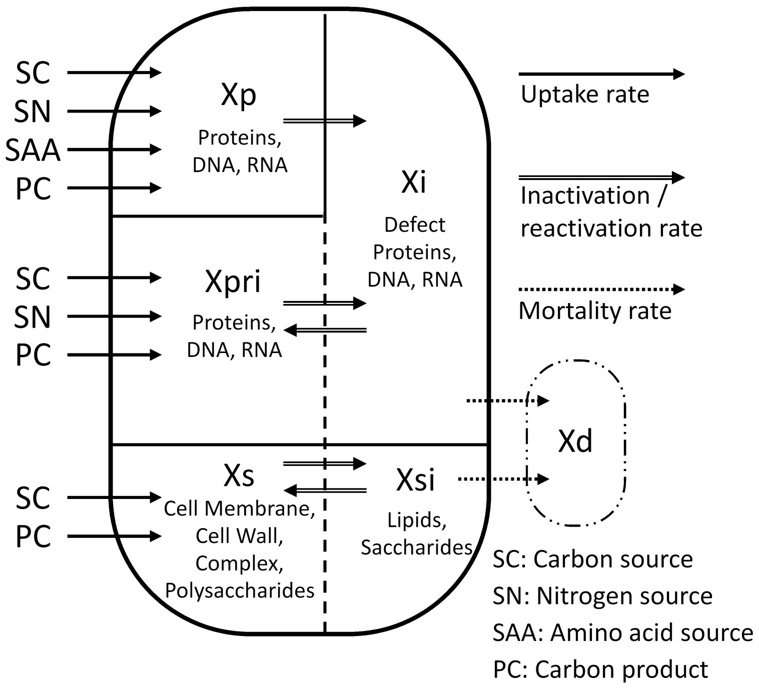

Fig. S1: Structure of the 6-compartment model, according to Brüning et al. (2017).

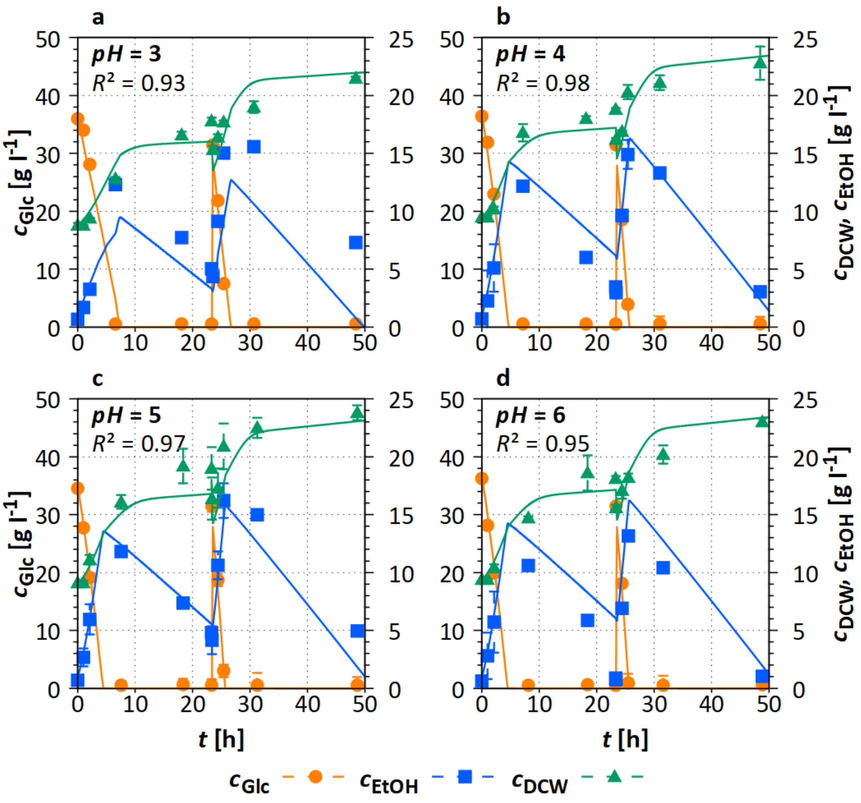

Fig. S2: Comparison of experimental data and simulated data for the adaption of pH-related model parameters. Error bars show standard deviation of two parallel shaking ask experiments for each investigated pH (4, 5, 6, 7, respectively) for biomass, glucose and ethanol concentrations. The quality of is represented by R^2 (optimal simulation x = y).

Tab. S1: Resulting median, 10% quantile and 90% quantile of the selected model parameters of the Monte Carlo-based uncertainty quantification for S1.

Model Parameter	Median	10% Quantile	90% Quantile
$r_{\mathrm{SC,mx,X}}$	$1.405E-04$	$1.079E-04$	$2.324E-04$
$r_{\mathrm{SN,mx,X}}$	$5.486E-05$	$4.966E-05$	$6.036E-05$
$r_{\mathrm{PC1,mx,X,Di}}$	$1.039E-05$	$9.606E-07$	$2.001E-05$
K_{act}	$1.073E-05$	$9.196E-06$	$1.138E-05$
K_{i}	$8.846E-05$	$6.238E-05$	$1.009E-04$
K_{d}	$5.123E-06$	$2.607E-06$	$8.045E-06$
$Y_{\mathrm{SC,X,1}}$	$8.091E-01$	$7.192E-01$	$8.965E-01$
$Y_{\mathrm{SC,X,2}}$	$1.484E-01$	$2.545E-02$	$4.460E-01$
$Y_{\mathrm{PC1,X}}$	$2.175E-01$	$7.362E-02$	$4.437E-01$
$Y_{\mathrm{PC2,X}}$	$2.457E-01$	$4.744E-02$	$5.364E-01$
$h500_{\mathrm{PC}}$	$6.845E+01$	$4.553E+01$	$1.403E+02$
$y_{\mathrm{Ch,0,PC}}$	$2.505E-01$	$1.057E-01$	$4.807E-01$
$xh50_{\mathrm{Ki,SN}}$	$2.014E+01$	$1.277E+01$	$2.315E+01$
$yh_{\mathrm{Ki,SN}}$	$9.920E+00$	$9.051E+00$	$1.064E+01$
$xl50_{\mathrm{Ki,SC}}$	$1.219E-02$	$3.341E-03$	$3.302E-02$
$yl_{\mathrm{Ki,SC}}$	$1.137E+00$	$1.003E+00$	$3.893E+00$
$h502_{\mathrm{SC}}$	$5.593E-01$	$2.704E-01$	$1.136E+00$
$y_{\mathrm{Ch,2,SC}}$	$5.007E-02$	$8.398E-03$	$1.289E-01$

3 Monte Carlo-based uncertainty quantification

3.1 Monte Carlo-based uncertainty quantification (S1)

The following model parameters have been adapted, see Subsection 2.5 in main manuscript:

- Uptake rates for glucose $r_{\mathrm{SC,mx,X}}$, nitrogen $r_{\mathrm{SN,mx,X}}$, ethanol $r_{\mathrm{PC1,mx,X,Di}}$ (Fig. S3 a, b, c)
- Activation K_{act} (Fig. S3d), inactivation K_{i}, mortality rates K_{d} (Fig. S3 d, e, f)
- Yield coefficients of glucose $Y_{\mathrm{SC,X,1}}$ & $Y_{\mathrm{SC,X,2}}$ (Fig. S4 a, b) and ethanol metabolic pathways $Y_{\mathrm{PC1,X}}$ & $Y_{\mathrm{PC2,X}}$ (Fig. S4 c, d)
- Ethanol inhibition boundary $h500_{\mathrm{PC}}$ and intensity $y_{\mathrm{Ch,0,PC}}$ (Fig. S4 e, f)
- Inhibition boundaries for very high concentration of nitrogen source $xh50_{\mathrm{Ki,SN}}$ & $yh_{\mathrm{Ki,SN}}$ (Fig. S5 a, b)
- Limitation $xl50_{\mathrm{Ki,SC}}$ & $yl_{\mathrm{Ki,SC}}$ (Fig. S5 c, d) and overlflow metabolism $h502_{\mathrm{SC}}$ & $y_{\mathrm{Ch,2,SC}}$ (Fig. S5 e, f) boundaries for glucose

A total of 18 model parameters (see Table S1) have been repeatedly identified for use with the mDoE-toolbox. The resulting median, 10% quantile and 90% quantile are shown in (see Table S1).

The resulting parameter distributions are depicted in Fig. S3 - S5.

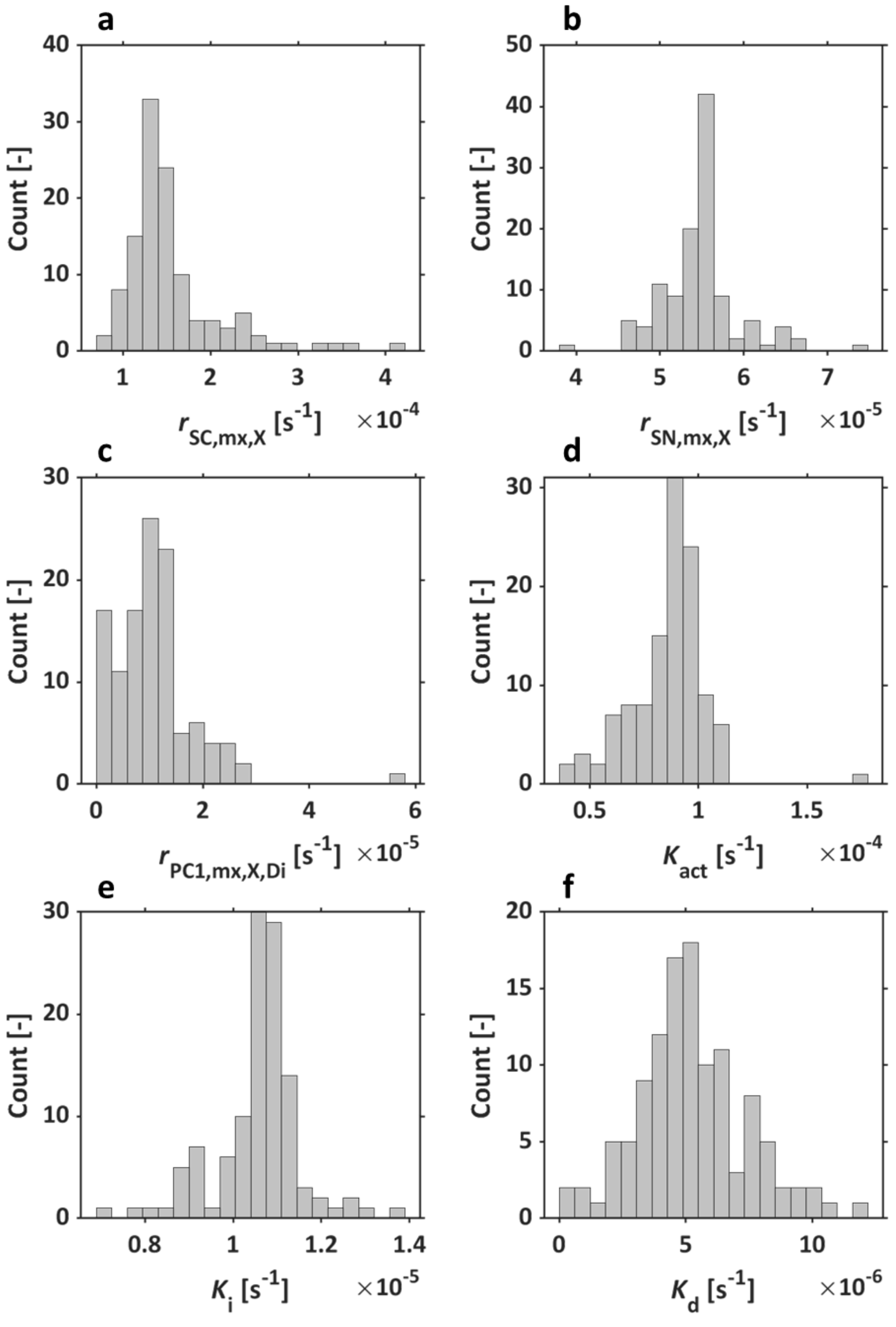

Fig. S3: Resulting parameter distributions of 116 adaptations for the uptake rates of glucose (a), nitrogen (b) and ethanol (c), as well as the activation (d), inactivation (e) and mortality rates (f), bins=20.

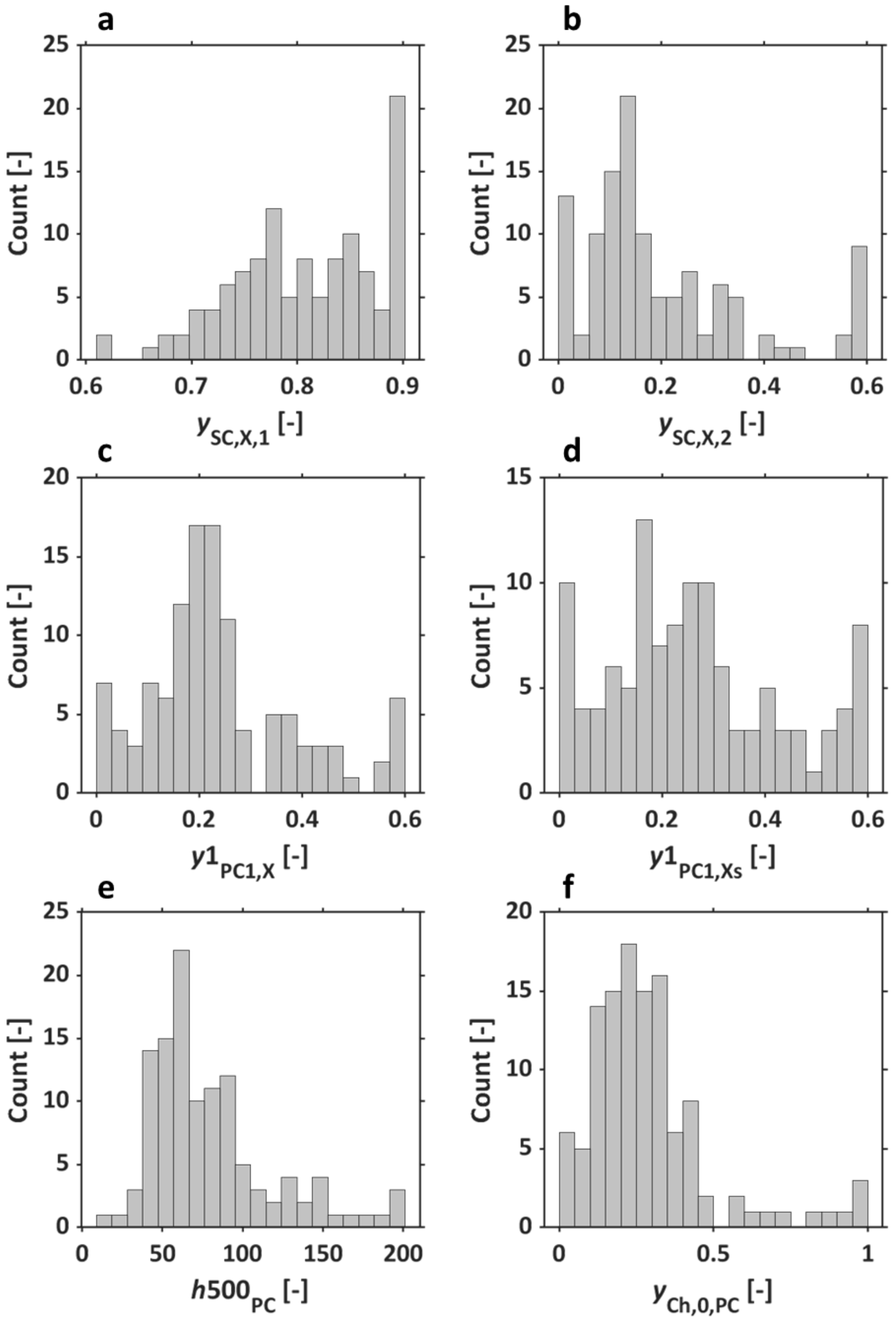

Fig. S4: Resulting parameter distributions of 116 parametrizations for the yield coefficients of the glucose (a & b) and ethanol metabolic pathways (c & d), as well as the ethanol inhibition boundary (e) and intensity (f), bins=20.

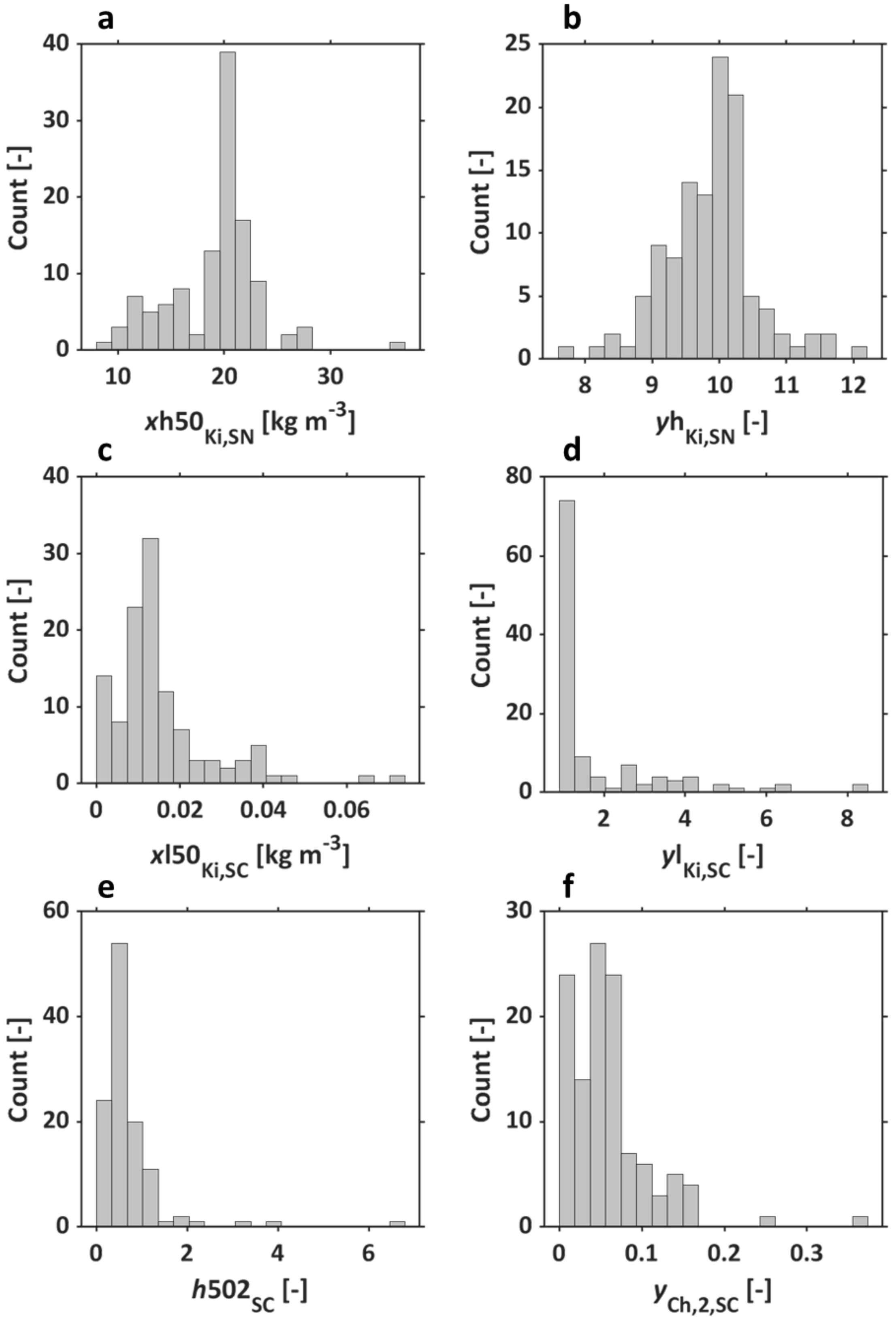

Fig. S5: Resulting parameter distributions of 116 parametrizations for the nitrogen source inhibition boundary (a) and intensity (b), as well as parameters for the glucose limitation (boundary (c) and intensity (d)) and overflow metabolism (crabtree effect) (boundary (e) and intensity (f)), bins=20.

Tab. S2: Resulting median, 10% quantile and 90% quantile of the selected model parameters of the Monte Carlo-based uncertainty quantification for S2.

Model Parameter	Median	10% Quantile	90% Quantile
$p_{\mathrm{BC,CO2}}$	$7.828E+01$	$5.190E+01$	$9.895E+01$
$Y_{\mathrm{PBC,SBC}}$	$5.910E-01$	$2.211E-01$	$8.484E-01$
$xh50_{\mathrm{Ki,SBC}}$	$7.637E-01$	$6.145E-01$	$1.459E+00$
$yh_{\mathrm{Ki,SBC}}$	$1.308E+01$	$1.080E+01$	$1.849E+01$
$K_{\mathrm{i,Xp}}$	$8.308E-06$	$5.385E-06$	$1.037E-05$
$r_{\mathrm{SC,mx,X}}$	$1.165E-05$	$8.489E-06$	$2.388E-05$
$r_{\mathrm{SN,mx,X}}$	$1.501E-04$	$1.035E-04$	$2.512E-04$
$r_{\mathrm{PC1,mx,X,Di}}$	$6.492E-06$	$1.504E-06$	$1.474E-05$
K_{act}	$6.771E-06$	$4.580E-06$	$9.435E-06$
K_{i}	$9.790E-05$	$9.418E-05$	$9.890E-05$
K_{d}	$3.732E-06$	$2.030E-06$	$6.022E-06$
$Y_{\mathrm{SC,X,1}}$	$3.106E-01$	$1.738E-01$	$4.563E-01$
$Y_{\mathrm{SC,X,2}}$	$8.896E-01$	$8.742E-01$	$8.979E-01$
$Y_{\mathrm{PC1,X}}$	$2.529E-01$	$1.255E-01$	$4.141E-01$
$Y_{\mathrm{PC2,X}}$	$2.799E-01$	$1.619E-01$	$4.448E-01$
$h500_{\mathrm{PC}}$	$8.348E+01$	$7.424E+01$	$9.044E+01$
$y_{\mathrm{Ch,0,PC}}$	$2.749E-01$	$1.113E-01$	$4.839E-01$
$h502_{\mathrm{SC}}$	$5.474E-01$	$2.471E-01$	$1.041E+00$
$y_{\mathrm{Ch,2,SC}}$	$1.079E-01$	$6.062E-02$	$1.600E-01$

3.2 Monte Carlo-based uncertainty quantification (S2)

The same model parameters as in S1 were adapted, but the inhibition boundaries of nitrogen and limitation of low glucose concentrations was removed because it had no effect on biocatalysis. Additionally, the parameters below were added for the description of the biocatalysis:

- Uptake rate for EAA $p_{\mathrm{BC,CO2}}$ (substrate biocatalysis S_BC) (Fig. S6 a)
- Selectivity of EAA to E3HB $Y_{\mathrm{PBC,SBC}}$ (product biocatalysis P_BC) (Fig. S6 b)
- Sigmoid parameters for the influence of high EAA concentration on the inactivation rate $xh50_{\mathrm{Ki,SBC}}$ & $yh_{\mathrm{Ki,SBC}}$ (Fig. S6 c, d)
- Inactivation rate for the biocatalytically active biomass $K_{\mathrm{i,Xp}}$ (Fig. S6 e)

A total of 19 model parameters (see Table S2) were repeatedly identified for use with the mDoE Toolbox. The resulting median, 10% quantile and 90% quantile are shown in (see Table S2).

The resulting parameter distributions are in Fig. S6 - S9.

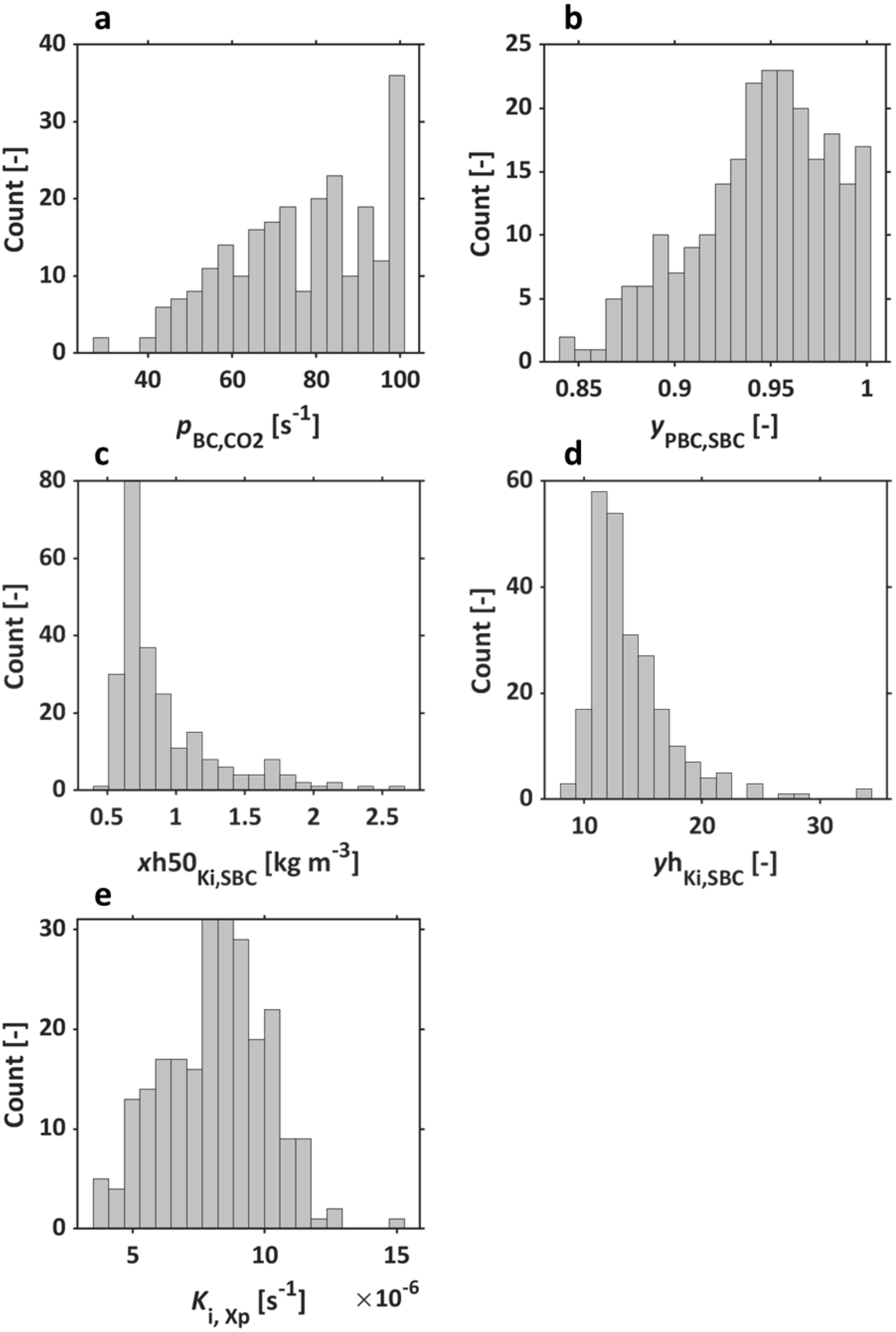

Fig. S6: Resulting parameter distribution of 240 parametrizations for the uptake rate of EAA (a), selectivity of EAA to E3HB (b), inhibition of high EAA concentration (c & d) and the inactivation rate of the biocatalytically active biomass (e), bins=20.

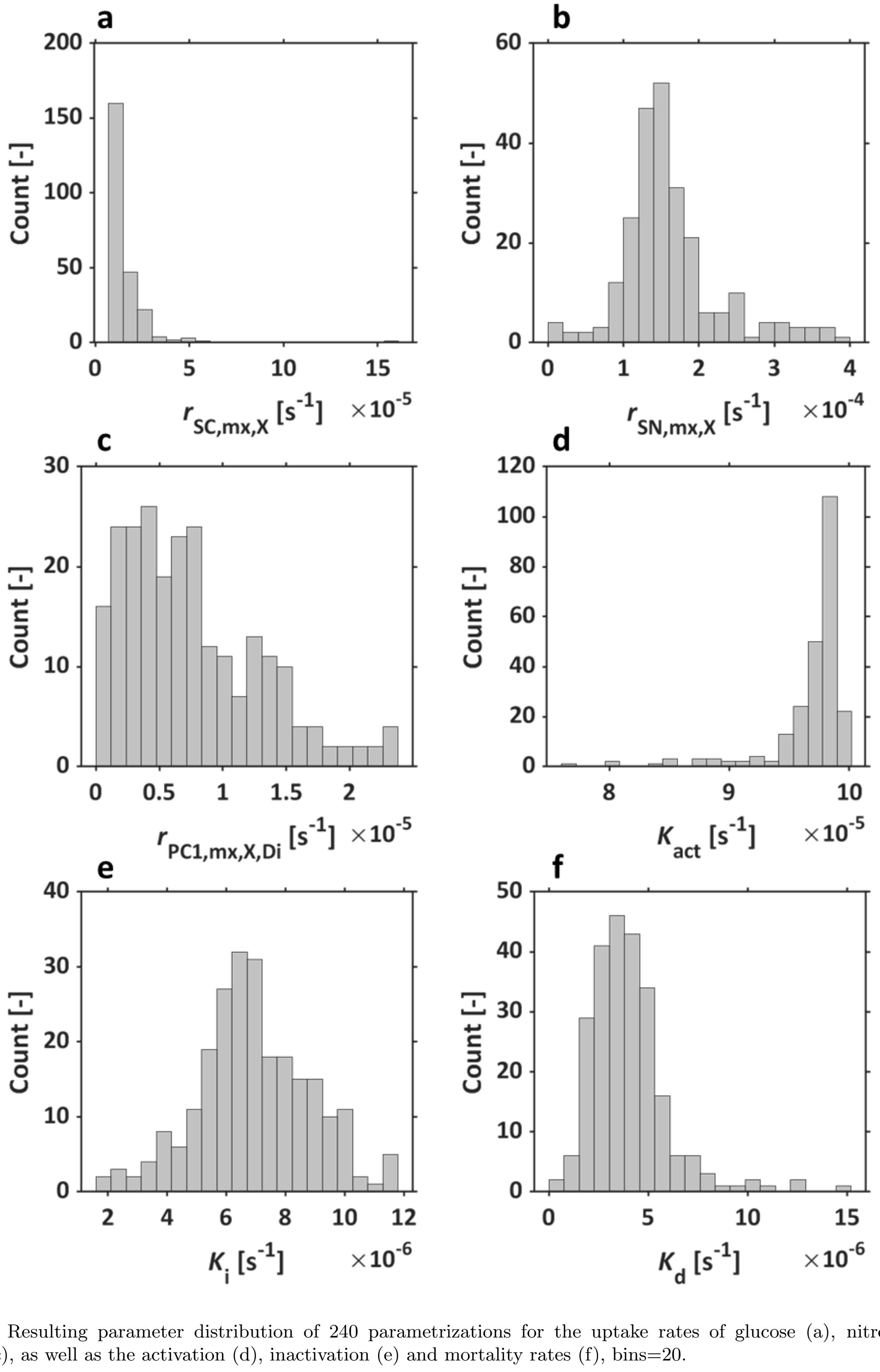

Fig. S7: Resulting parameter distribution of 240 parametrizations for the uptake rates of glucose (a), nitrogen (b) and ethanol (c), as well as the activation (d), inactivation (e) and mortality rates (f), bins=20.

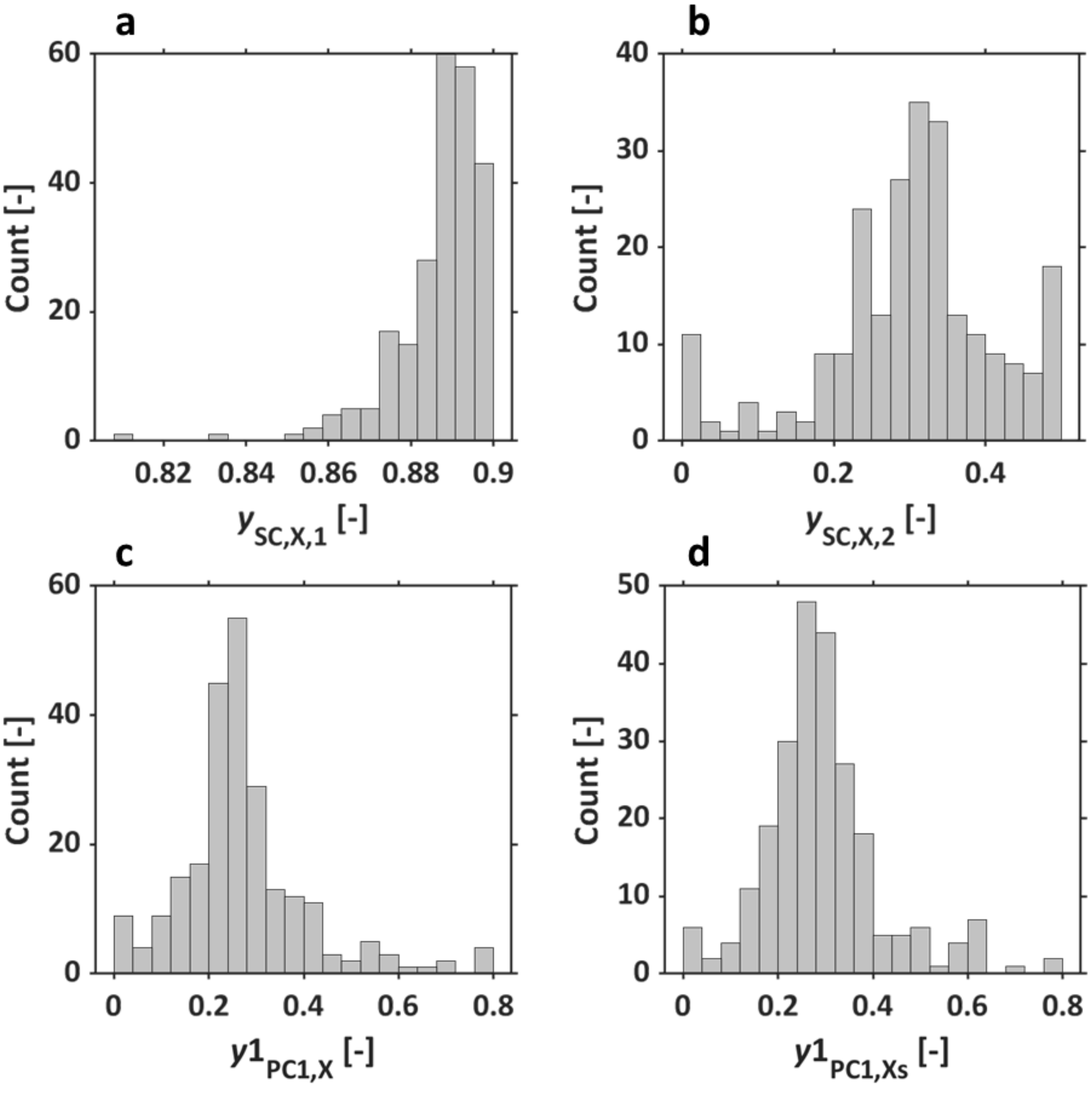

Fig. S8: Resulting parameter distribution of 240 parametrizations for the yield coefficients of the glucose (a & b) and ethanol metabolic pathways (c & d), bins=20.

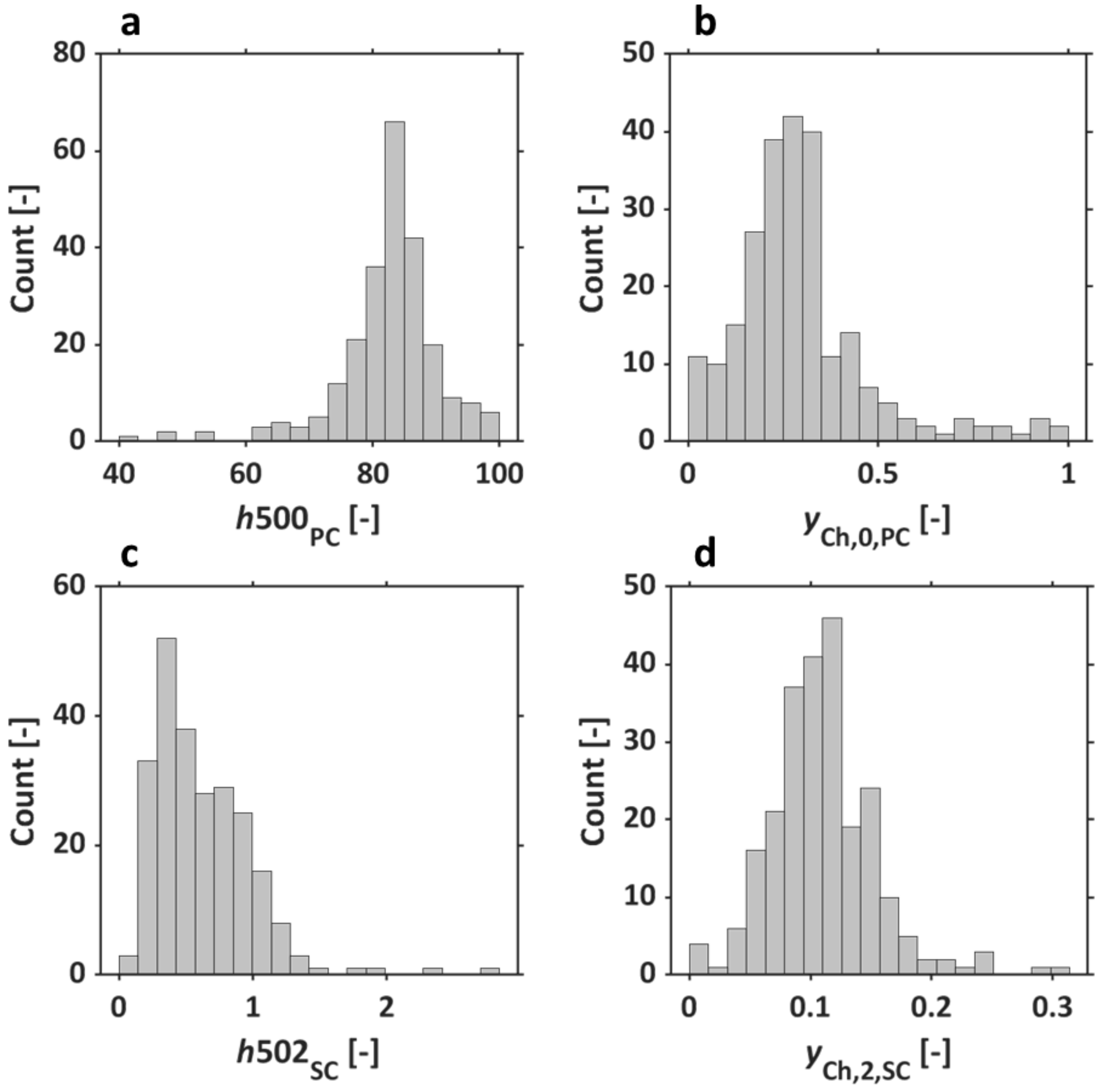

Fig. S9: Resulting parameter distribution of 240 parametrizations for the ethanol inhibition boundary (a) and intensity (b), as well as the glucose overflow metabolism (crabtree effect) boundary (c) and intensity (d), bins=20.

4 Optimization of fed-batch process with mDoE toolbox (S1)

Fig: S10 shows the online data of the off-gas measurement and the calculated values for volume (V) and feed (F).

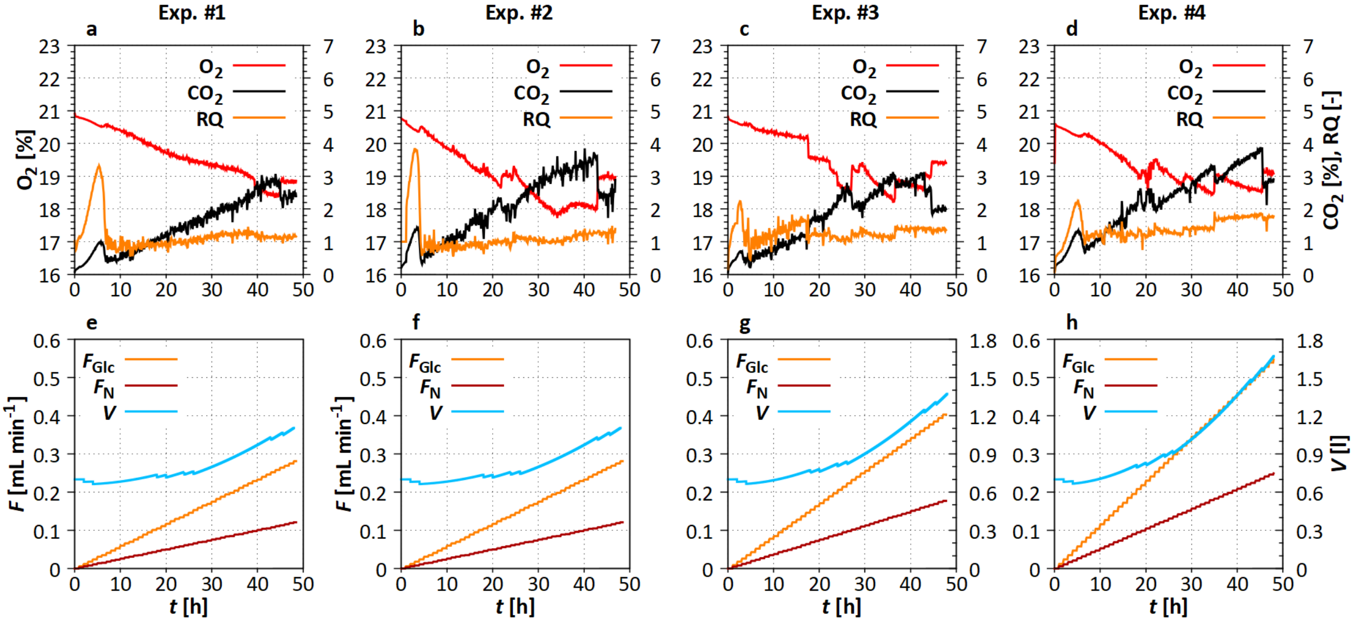

Fig. S10: Supplementary data for Fig: 6. Online data of the off-gas measurement as well as the calculated respiratory quotient (a-d). Calculated V and F (e-f)

5 Quadratic functions

For the Response surfaces, quadratic functions with the following structure were used.

$$f(x,y,z) = a_1x^2 + a_2y^2 + a_3z^2 + b_1xy + b_2xz + b_3yz + c_1x + c_2y + c_3z + d$$

The parameters of the quadratic function for yeast cultivation and biocatalysis are listed Table S3.

Tab. S3: Parameter of quadratic function for the high cell density process (S1) and the biocatalysis (S2).

**Parameter**	**S1**	**S2**
a_1	-0.129	0.407
a_2	0.615	2.45
a_3	2.43	14.4
b_1	0.506	-2.04
b_2	0.627	104
b_3	0.0218	16.2
c_1	0.0992	26.0
c_2	0.165	-21.8
c_3	1.54	-654
d	-0.0573	-85.3

Nomenclature

Variable	Explanation	Unit
$a_\mathrm{i}, b_\mathrm{i}, c_\mathrm{i}, d$	parameters of quadratic response surface function	[-]
c_k	concentration of component k in the bioreactor	[g l^{-1}]
F_k	feeding rate of component k	[ml min^{-1}]
k	EAA, Glc, N, EtOH, E3HB	[-]
K_act	reactivation rate	[s^{-1}]
K_d	mortality rate	[s^{-1}]
K_i	inactivation rate	[s^{-1}]
K_s	Saturation constant	[g l^{-1}]
K_s1	gradient slope	[-]
PC	carbon product	[g l^{-1}]
r_{c_s}	total uptake / consumption rate	[h^{-1}]
r_S	substrate uptake rate	[g $\mathrm{l}^{-1}\mathrm{h}^{-1}$]
R^2	coefficient of determination	[-]
SBC	biocatalysis educt	[g l^{-1}]
SC	carbon substrate	[g l^{-1}]
SN	nitrogen substrate	[g l^{-1}]
t	time	[h]
V	Volume	[l]
x, y, z	Dimensions	[-]
$X_\mathrm{50,l}$	low location parameter of x	[-]
$X_\mathrm{50,h}$	high location parameter of x	[-]
Xd	dead biomass	[g l^{-1}]
Xi	inactive biomass	[g l^{-1}]
Xp	product forming biomass	[g l^{-1}]
Xpri	autocatalytically active biomass	[g l^{-1}]
Xs	structurally active biomass	[g l^{-1}]
Xsi	structurally inactive biomass	[g l^{-1}]
X_v	viable Biomass	[g l^{-1}]
y_k	measured value	[-]
$Y_\mathrm{k/S}$	yield coefficient	[-]

Abbreviations

Abbreviation	Explanation
DCW	dry cell weight
DoE	design of experiments
DO	dissolved oxygen
EAA	ethyl acetoacetate
EtOH	ethanol
E3HB	(S)-ethyl-3-hydroxybutyrate
Exp.	experiment
Glc	glucose
mDoE	model-assisted design of experiments
RQ	respiratory quotient

References

Brüning, S., Gerlach, I., Pörtner, R., Mandenius, C.-F., and Hass, V. C. (2017). Modeling suspension cultures of microbial and mammalian cells with an adaptable six-compartment model. *Chemical Engineering & Technology*, 40(5):956–966.
